# Using a longitudinal network structure to subgroup depressive symptoms among adolescents

**DOI:** 10.1186/s40359-024-01537-8

**Published:** 2024-01-24

**Authors:** Sugai Liang, Zejun Huang, Yiquan Wang, Yue Wu, Zhiyu Chen, Yamin Zhang, Wanjun Guo, Zhenqing Zhao, Sabrina D. Ford, Lena Palaniyappan, Tao Li

**Affiliations:** 1 Affiliated Mental Health Centre & Hangzhou Seventh People’s Hospital, Zhejiang University School of Medicine, 305 Tianmushan Road, 310013 Hangzhou, China; 2 Hangzhou Institute of Educational Science, 310003 Hangzhou, China; 3 Hangzhou Vocational & Technical College, 310018 Hangzhou, China; 4 Douglas Mental Health University Institute, Department of Psychiatry, McGill University, H4H1R3 Montreal, Canada; 5 Robarts Research Institute, Schulich School of Medicine and Dentistry, Western University, N6A5K8 London, Canada; 6 Department of Medical Biophysics, Western University, N6A5K8 London, Canada; 7 Liangzhu Laboratory, MOE Frontier Science Center for Brain Science and Brain-machine Integration, State Key Laboratory of Brain-machine Intelligence, Zhejiang University, 310000 Hangzhou, China; 8 NHC and CAMS Key Laboratory of Medical Neurobiology, Zhejiang University, 310063 Hangzhou, China

**Keywords:** Adolescent, Depressive symptoms, Anxiety, Network analysis

## Abstract

**Background:**

Network modeling has been proposed as an effective approach to examine complex associations among antecedents, mediators and symptoms. This study aimed to investigate whether the severity of depressive symptoms affects the multivariate relationships among symptoms and mediating factors over a 2-year longitudinal follow-up.

**Methods:**

We recruited a school-based cohort of 1480 primary and secondary school students over four semesters from January 2020 to December 2021. The participants (*n* = 1145) were assessed at four time points (ages 10–13 years old at baseline). Based on a cut-off score of 5 on the 9-item Patient Health Questionnaire at each time point, the participants were categorized into the non-depressive symptom (NDS) and depressive symptom (DS) groups. We conducted network analysis to investigate the symptom-to-symptom influences in these two groups over time.

**Results:**

The global network metrics did not differ statistically between the NDS and DS groups at four time points. However, network connection strength varied with symptom severity. The edge weights between learning anxiety and social anxiety were prominently in the NDS group over time. The central factors for NDS and DS were oversensitivity and impulsivity (3 out of 4 time points), respectively. Moreover, both node strength and closeness were stable over time in both groups.

**Conclusions:**

Our study suggests that interrelationships among symptoms and contributing factors are generally stable in adolescents, but a higher severity of depressive symptoms may lead to increased stability in these relationships.

**Supplementary Information:**

The online version contains supplementary material available at 10.1186/s40359-024-01537-8.

## Background

Depression in adolescents is a serious public health concern [1, 2] that is associated with various negative outcomes, such as loss of productivity, psychosocial dysfunction and suicide, contributing to a substantial burden of disability [3]. Additionally, depression in early life has long-lasting impacts, including increased risks for adulthood depression, anxiety and substance abuse, as well as worse health status [4, 5]. Increasing evidence suggests that depressive disorder is a continuum with features of emotional distress, including low mood, irritability, impulsivity, helplessness and other symptoms [6, 7]. Similar to clinically diagnosed depression, depressive symptoms (DSs) in adolescents are linked to social functioning problems, impaired educational attainment and poor outcomes in adulthood [8, 9]. Despite the frequent occurrence and significant consequences of depressive symptoms, symptoms are often overlooked by families, teachers and clinicians [10, 11]. China has initiated a national program to screen for depression in school-going children [12], highlighting the global importance of understanding the features that contribute to the persistence of DSs in adolescents and their later evolution into a depressive disorder.

Depression and anxiety symptoms are highly interconnected from early childhood through mid-adolescence [13]. In addition, childhood anxiety is associated with the childhood-persistent trajectory of DSs [5]. Moreover, several aspects of anxiety in childhood, such as physiological reactivity, worry/oversensitivity, social anxiety and alienation, can influence mental health outcomes in adolescence in the presence of a depressive burden [14, 15]. Furthermore, contextual factors, such as a lack of friendships and poor home (family) environment [16], and behavioral factors, such as impulsivity [17], loss of temper [18] and low help-seeking behaviors [19], are significant in predicting persistent depression in adolescents. Taken together, these results show that the consolidation effect of DSs to a syndrome needing intervention depends on the various contextual and behavioral factors described above, as well as the emergence of comorbidities, especially anxiety.

## Current study

Symptom network studies offer a systems perspective of phenomenological features and allow us to probe how contextual and behavioral factors relate to a system [20]. While network analysis has been applied to depressive burden and anxiety symptoms in a cross-sectional manner in previous studies [13, 21–23], this approach has not been used to study the longitudinal trajectory of DSs among adolescents to date. Longitudinal sampling in an untreated community-based sample will allow us to examine how the relationship among depression, anxiety and contextual and behavioral factors affecting mental health operates over time in adolescents. One promising aspect of the network approach is that it can gain insight into intervention strategies by identifying the central symptoms that contribute to most of the complex interactions; these symptoms tend to carry a higher overall importance than other symptoms in a network [13, 22].

In the present study, we investigated DSs and related factors (comorbid anxiety, contextual and behavioral factors) at the network level among adolescent students over 4 time points with a 2-year longitudinal cohort design. We hypothesized that the interrelationship among anxiety symptoms, contextual factors and behavioral factors indicating symptom persistence would be stronger in adolescents with DSs than in those with non-depressive symptoms (NDSs). We also anticipated that the consolidating effect of depression on behavioral factors and anxiety would be seen across multiple time points in a school-based cohort during a time of macrosystem disruption (SARS-CoV-2 pandemic phase, 2020-21). On the other hand, adolescents without notable depressive symptoms could still experience anxiety-related symptoms but would not show a consolidation effect involving contextual and behavioral factors.

## Methods

### Study sample

Data were collected from primary and secondary school students through a school-level survey administered at the beginning of every spring and fall semester from January 2020 to December 2021. In this study, entire cohorts of 4th, 5th, 6th and 7th grade students aged 10–13 years were recruited for the initial assessment. The total sample included 1480 participants. We collected data across four time points, including t1 (baseline), t2, t3 and t4. The interval between the two assessments was half a year. Informed parental consent was required before adolescents could be invited to participate. This study was carried out in accordance with the Declaration of Helsinki and was approved by the local research ethics committee.

### Measures

The survey was administered by the study team and trained teachers during school hours in a computer room. Demographic information, including age, sex and grade, was recorded. The 9-item Patient Health Questionnaire (PHQ-9) was used to measure the presence and severity of depressive symptomatology [24]. A score of more than or equal to 5 indicated that a participant had DSs [25]. In this study, participants with a PHQ-9 score below 5 at baseline and each follow-up were included in the NDS group, and those with a score equal to or above 5 were included in the DS group.

The Mental Health Test (MHT) was used to assess general anxiety on 8 dimensions, including learning anxiety, social anxiety, loneliness, self-blaming tendencies, oversensitivity, somatic anxiety, phobia anxiety and impulsive tendencies [26]. A score of greater than 8 on any subscale or a total score of more than 56 is considered to indicate high risk of psychological problems. In addition, it was included six additional questions regarding the following to capture factors that affect depressive burden in this age group: family environment, friendship, help-seeking behaviors, loss of temper, hopelessness, and lying. The questions were rated on a five-point scale, and the two items for hopelessness and lying were reverse scored (see online Supplementary Information for details). Participants (*n* = 1145) who completed the self-report scales at all four time points were included in the analysis. The data screening flowchart is shown in Supplementary Fig. S1.

### Statistical analysis

The chi-square test was used to compare sex and DS frequency over time, and the Wilcoxon rank sum t test was used to compare scale scores between the NDS and DS groups. The Kruskal‒Wallis test was applied to compare scale scores between time points in each group. A probability level of *p* <.05 was considered statistically significant. Effect size was calculated using Cohen’s d. The Jaccard similarity coefficient, a measure of the similarities between sample sets [27], was calculated for each group across the four time points.

### Network construction

All analyses were conducted using R (Version 4.1.3) [28]. A Gaussian graphical model (GGM) [29] was used to fit the data to understand conditional dependency relationships between variables. Based on different feature dimensions, including depressive symptom levels (PHQ-9 as a single score, given its one-factor fit in Chinese adolescents [30]), mediating factors and anxiety features, networks were constructed using 14 nodes [PHQ-9 score, Learning anxiety (Learn), Social anxiety (Soc), Loneliness (Lon), Self-blaming (Blame), Worry and Oversensitivity (Sen), Somatic anxiety (Som), Phobia (Pho), Impulsivity (Imp), Family environment (FE), Friendship (FS), Loss of temper (LT), Hopelessness (HL), and Help-seeking behavior (HS)] for the two groups at each time point. The edge weights of the GGM were computed by partial correlations. Gaussian Markov random fields were applied to learn the graphical structure, and graphical LASSO [31] and the extended Bayesian information criterion (EBIC) model [32] were used to select the optimal regularization parameter. For the EBIC model, the tuning hyperparameter (*γ*) was set to 0.5, which yields accurate network estimations [33]. The network layouts were computed and derived from the Fruchterman–Reingold algorithm [34].

### Network analysis

#### Global network metrics

Global network metrics consisting of the network density, global strength, average clustering coefficient and characteristic path length were calculated using the ‘qgraph’ [35] and ‘igraph’ [36]. The network density was the ratio of the number of edges to the number of all possible edges, and global strength was defined as the weighted absolute sum of all edges in a given network [37]. The clustering coefficient was defined as the probability that the adjacent nodes of a node were connected in a graph, and characteristic path length was the average shortest path length between all pairs of nodes in the network. In network comparisons, the R package ‘NetworkToolbox’ [38] was used to investigate the differences in the network density, average clustering coefficient and characteristic path length between networks. The ‘Network Comparison Test (NCT)’ [39] was applied to compare the invariance of global strength between networks using a resampling-based permutation test and randomly repeated 1000 times.

#### Local network metrics

Given that previous research suggested that betweenness and closeness centrality seem unsuitable as measures of node importance for psychological networks [40, 41], in this study, node strength was calculated for the centrality index. In the ‘qgraph’ procedure [35], the node strength was defined as the sum of z scores of absolute weights connected to the central node. Additionally, the NCT [39] was used to compare the invariance of the network structure and node strength among the networks. If the network structure exhibited significant differences, the specific edges were calculated and identified.

#### Network accuracy and stability

The accuracy and stability of the network were estimated using the ‘bootnet’ [42]. For the nonparametric bootstrapped networks, 1000 iterations were run to estimate 95% confidence intervals (CIs) for the accuracy of edge weights, and the case-dropping subset bootstrapping networks were calculated to investigate the order stability of node centrality indices. In addition, the correlation stability coefficient (CS-coefficient) was calculated to measure the stability of node centrality indices [42]. The correlation between the original centrality indices based on the full data was compared to the correlation obtained from the subset of data representing different percentages of the overall sample. The CS-coefficient should be above 0.25 and, preferably, above 0.5. Finally, bootstrapped difference tests were performed on edge weights and centrality indices to test whether they differed significantly from each other.

## Results

### Sample characteristics

Among the 1480 eligible students, 1145 participants (585 [51.1%] girls) had valid data at the four time points, including 277 4th grade students (24.2%; average age 10 years), 242 5th grade students (21.1%; average age 11 years), 258 6th grade students (22.5%; average age 12 years) and 368 7th grade students (32.1%; average age 13 years). At the four time points, the percentages of PHQ-9 scores equal to or above 5 were 27.9%, 27.9%, 32.2% and 31.2%, respectively. There were no significant changes in DS frequency over time (*p* =.05). The total and subscale scores of the MHT were significantly lower in the NDS group than in the DS group over time (Table 1). No significant differences were found in the PHQ-9 scores between t1 and every follow-up in any group (Supplementary Table S1). Supplementary Fig. S2 illustrates the heatmap of the Pearson correlation matrix among all fourteen variables across the entire sample. The Jaccard similarity coefficient was 0.20 and 0.53 in the DS and NDS groups, respectively, at all four time points.

**Table 1:** Psychological manifestations in the NDS and DS groups at baseline and follow-ups

Characteristics	Baseline (t1)		t2		t3		t4	
	NDS	DS	NDS	DS	NDS	DS	NDS	DS
	*N* = 825	*N* = 320	*N* = 825	*N* = 320	*N* = 776	*N* = 369	*N* = 788	*N* = 357
Age, median (IQR), y^a^	12 (11, 13)	12 (10, 13)			13 (11, 14)	13 (12, 14)		
Sex (Girls/Boys)^b^	421/404	164/156	426/399	159/161	384/392	201/168	397/391	188/169
PHQ-9^c^	1 (0–2)	7 (6–10)	1 (0–2)	7 (6–10)	1 (0–3)	8 (6–10)	1 (0–2)	7 (6–9)
General MHT^c^	19 (11–29)	44 (33.75-54)	18 (10–28)	43.5 (31–54)	19 (11–30)	46 (35–56)	18 (9–27)	44 (32–55)
Learning anxiety^c^	5 (3–8)	10 (7–12)	5 (3–8)	10 (7–12)	6 (4–9)	11 (8–12)	5 (3–8)	10 (7–12)
Social anxiety^c^	2 (1–4)	5 (4–7)	2 (1–4)	5 (4–7)	3 (1–4)	6 (4–8)	2 (1–4)	6 (4–7)
Loneliness^c^	1 (0–2)	3 (1–5)	1 (0–2)	2 (1–4)	1 (0–2)	3 (1–5)	0 (0–1)	3 (1–5)
Self-blaming^c^	3 (1–5)	6 (3–8)	2 (1–4)	5.5 (3–8)	3 (1–5)	6 (4–8)	2 (0–4)	5 (3–8)
Oversensitivity^c^	3 (1–5)	6 (5–8)	3 (1–5)	6 (5–8)	3 (2–5)	7 (6–8)	3 (1–5)	7 (5–8)
Somatic anxiety^c^	2 (1–3)	6 (4–8)	2 (1–4)	6 (3–8)	2 (1–4)	7 (4–9)	2 (0–3)	6 (4–9)
Phobia anxiety^c^	1 (0–3)	4 (2–6)	1 (0–3)	4 (1–6)	0.5 (0–2)	3 (1–5)	0 (0–2)	3 (1–5)
Impulsivity^c^	0 (0–2)	4 (2–6)	0 (0–1)	3 (2–6)	0 (0–1)	4 (2–6)	0 (0–1)	3 (2–6)

Scale data were expressed in median (IQR). IQR, interquartile range (Q1-Q3). NDS, nondepressive symptom group. DS, depressive symptom group. PHQ-9, 9-item Patient Health Questionnaire. MHT, Mental Health Test. ^a^ comparison between the NDS and DS groups by Wilcoxon rank sum t test, *P* >.05 at t1 and t3. ^b^ comparison between the NDS and DS groups by chi-square, *P* >.05 at four time points. ^c^ comparison between the NDS and DS groups by Wilcoxon rank sum t test, *P* <.001 at four time points

^a^comparison between the NDS and DS groups by Wilcoxon rank sum t test, *P* >.05 at t1 and t3

^b^comparison between the NDS and DS groups by chi-square, *P* >.05 at four time points

^c^comparison between the NDS and DS groups by Wilcoxon rank sum t test, *P* <.001 at four time points

### Global network metrics

**Fig. 1:**
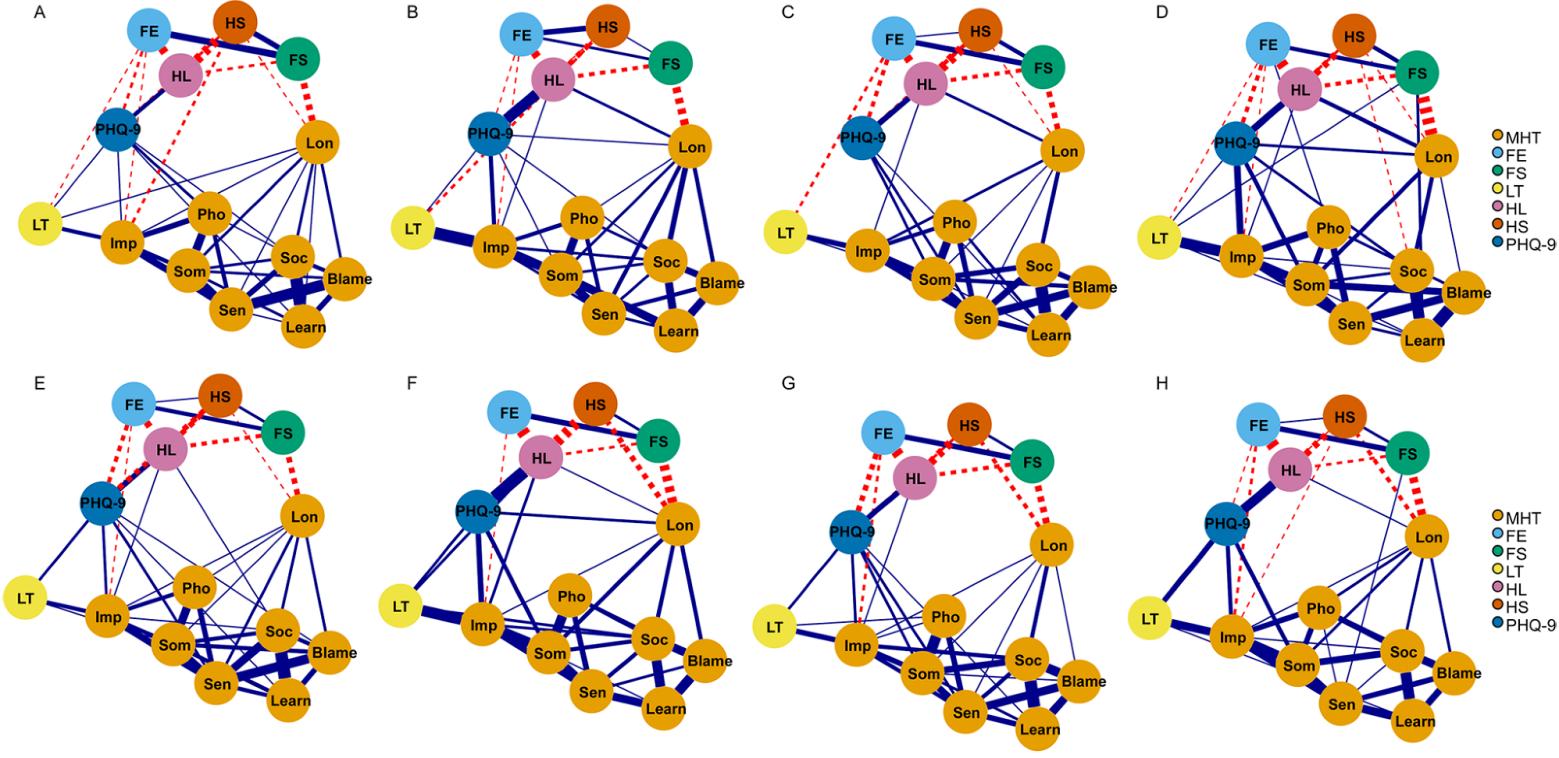
Estimated network structures. (**A**) The non-depressive symptom (NDS) group at t1 (baseline). (**B**) The depressive symptom (DS) group at t1 (baseline). (**C**) The NDS group at t2. (**D**) The DS group at t2. (**E**) The NDS group at t3. (**F**) The DS group at t3. (**G**) The NDS group at t4. (**H**) The DS group at t4. The names of the nodes (‘Learn’, ‘Soc’, ‘Lon’, ‘Blame’, ‘Sen’, ‘Som’, ‘Pho’, ‘Imp’, ‘FE’, ‘FS’, ‘LT’, ‘HL’, ‘HS’ and ‘PHQ-9’) were ‘Learning anxiety’, ‘Social anxiety’, ‘Loneliness’, ‘Self-blaming’, ‘Oversensitivity’, ‘Somatic anxiety’, ‘Phobia’, ‘Impulsivity’, ‘Family environment’, ‘Friendship’, ‘Loss of temper’, ‘Hopelessness’, ‘Help-seeking’ and ‘PHQ-9 score’. MHT, mental health testing. PHQ-9, the 9-item Patient Health Questionnaire. Red dashed edges indicate negative weights, and blue edges indicate positive weights

The resulting network was well connected, with no isolated nodes (Fig. 1). There were no significant differences in the global network metrics between the NDS and DS groups at the four time points (Table 2), except for a trend of higher global strength in the DS group than the NDS group at t2 and t3 (*p* =.05). This indicates that the presence of a higher DS burden does not alter the overall risk-symptom relationship per se.

**Table 2:** Global connectivity of the networks in the NDS and DS groups at baseline and follow-ups

Characteristics	Baseline (t1)			t2			t3			t4			Average of t1 to t4		
	NDS	DS	P^a^	NDS	DS	P^a^	NDS	DS	P ^a^	NDS	DS	P^a^	NDS	DS	Cohen’s d
Density	0.56	0.50	0.13	0.54	0.51	0.55	0.51	0.50	0.93	0.52	0.50	0.66	0.53	0.50	1.87
Global strength	5.63	5.68	0.81	5.59	6.16	0.05	5.24	5.73	0.05	5.54	5.82	0.16	5.50	5.85	1.76
Average clustering coefficient	0.70	0.70	0.99	0.64	0.63	0.97	0.73	0.71	0.71	0.73	0.67	0.31	0.70	0.68	0.57
Average shortest path length	1.35	1.44	0.28	1.37	1.41	0.67	1.41	1.44	0.67	1.40	1.42	0.70	1.38	1.43	2.03

^a^is the results of permutation test (global strength from NCT and the others from NetworkToolbox). NDS, nondepressive symptom group. DS, depressive symptom group

### Local network metrics

The top three positive edge weights for the NDS group were almost the same over time and comprised edges between learning anxiety and social anxiety, between somatic anxiety and phobia, and between self-blaming and oversensitivity (at t1, t2 and t3). The link between social anxiety and self-blaming was also prominent at t4 in the NDS group. The top three positive edge weights for the DS group differed over time and included edges between impulsivity and loss of temper, between the PHQ-9 score and hopelessness, among items related to impulsivity, somatic anxiety, learning anxiety, self-blaming and social anxiety in the MHT (Fig. 1). This indicates that DSs influence the covariance among anxiety and externalizing behaviors, and may introduce a degree of instability within the symptom network. The weights of all edges in both groups at the four time points are listed in Tables S2–S9.

In NCT analysis, the network structure invariance test revealed a significant difference in edge weights (*p* =.03) between the NDS and DS groups only at t1. The particular edges that differed across the two networks were investigated and identified with Bonferroni‒Holm correction. The edge weights between impulsivity and loss of temper were statistically significant, and at t1, the DS group manifested higher strength between impulsivity and loss of temper than the NDS group (0.33 versus 0.12).

**Fig. 2:**
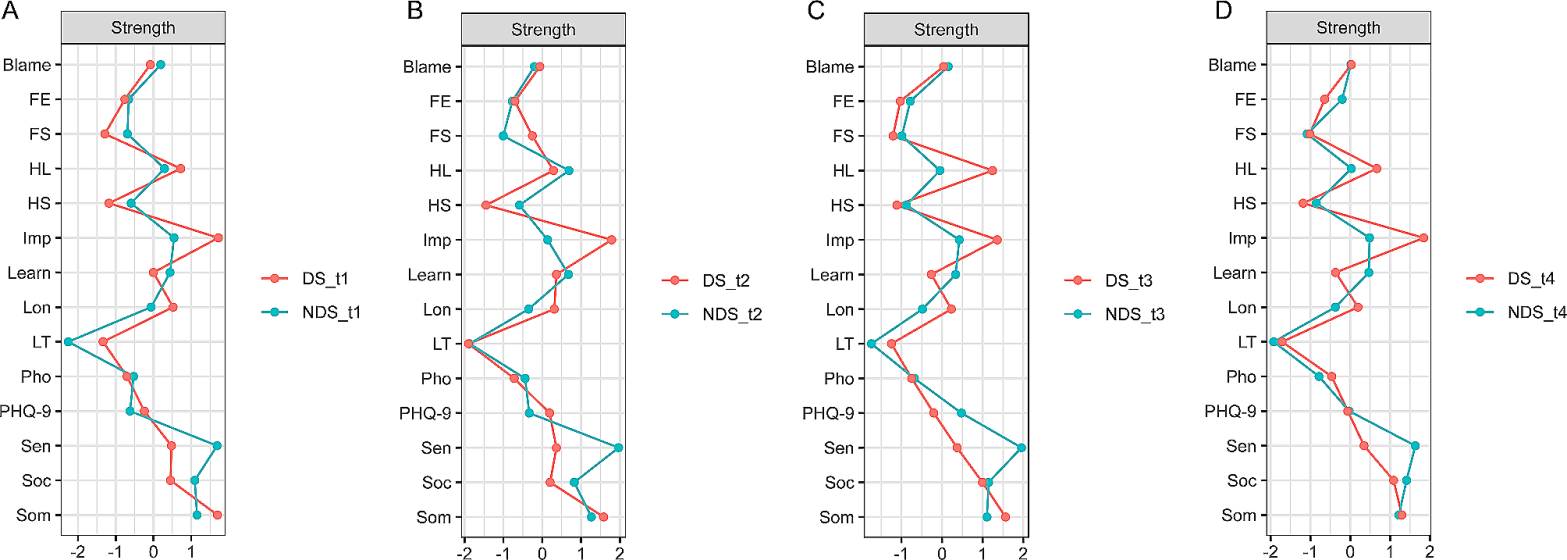
Centrality index of nodal strength. (**A**) The depressive symptom and non-depressive symptom groups at baseline (DS-t1 and NDS-t1). (**B**) The depressive symptom and non-depressive symptom groups at t2 (DS-t2 and NDS-t2). (**C**) The depressive symptom and non-depressive symptom groups at t3 (DS-t3 and NDS-t3). (**D**) The depressive symptom and non-depressive symptom groups at t4 (DS-t4 and NDS-t4). The centrality index is shown as standardized z scores. The names of the nodes (‘Blame’, ‘FE’, ‘FS’, ‘HL’, ‘HS’, ‘Imp’, ‘Learn’, ‘Lon’, ‘LT’, ‘Pho’, ‘PHQ-9’, ‘Sen’, ‘Soc’, ‘Som’) were ‘Self-blaming’, ‘Family environment’, ‘Friendship’, ‘Hopelessness’, ‘Help-seeking’, ‘Impulsivity’, ‘Learning anxiety’, ‘Loneliness’, ‘Loss of temper’, ‘Phobia’, ‘PHQ-9 score’, ‘Oversensitivity’, ‘Social anxiety’, and ‘Somatic anxiety’. PHQ-9, the 9-item Patient Health Questionnaire

In terms of the centrality index, oversensitivity had the highest node strength for the networks of the NDS group over time. The items with the highest node strength for the networks of the DS group were impulsivity at t1, t2 and t4 and somatic anxiety at t3 (Fig. 2). In the NDS group, the average node strength between follow-up and baseline was greatest for oversensitivity. In the DS group, the average node strength was greatest for impulsivity (see Table 3). Oversensitivity and impulsivity had the largest effect-size differences between the NDS and DS groups among the four time points. This was consistent with the results of network comparison tests for node strength between the two groups from t1 to t4 (Table S10).

**Table 3:** Nodal strength values in the NDS and DS groups from t1 to t4

Node	t1		t2		t3		t4		Average of t1 to t4		
	NDS	DS	NDS	DS	NDS	DS	NDS	DS	NDS	DS	Cohen’s d
Blame	0.19	-0.08	-0.20	-0.06	0.15	0.04	0.02	0.03	0.04	-0.02	0.44
FE	-0.66	-0.76	-0.77	-0.70	-0.78	-1.03	-0.20	-0.64	-0.60	-0.78	0.79
FS	-0.69	-1.29	-1.00	-0.26	-0.99	-1.21	-1.08	-1.01	-0.94	-0.94	0.01
HL	0.29	0.72	0.69	0.29	-0.05	1.24	0.03	0.66	0.24	0.73	1.34
HS	-0.59	-1.18	-0.59	-1.45	-0.88	-1.11	-0.85	-1.19	-0.73	-1.23	3.27
Imp	0.54	1.72	0.14	1.79	0.42	1.36	0.48	1.84	0.40	1.68	6.47
Learn	0.44	0.00	0.68	0.37	0.33	-0.26	0.47	-0.37	0.48	-0.07	2.14
Lon	-0.06	0.52	-0.35	0.31	-0.48	0.23	-0.38	0.19	-0.32	0.31	3.83
LT	-2.26	-1.34	-1.87	-1.90	-1.74	-1.24	-1.92	-1.71	-1.95	-1.55	1.48
Pho	-0.53	-0.70	-0.44	-0.72	-0.68	-0.75	-0.79	-0.47	-0.61	-0.66	0.35
PHQ-9	-0.62	-0.24	-0.33	0.19	0.48	-0.20	-0.03	-0.06	-0.13	-0.08	0.13
Sen	1.69	0.48	1.97	0.36	1.96	0.37	1.63	0.34	1.81	0.39	10.68
Soc	1.09	0.45	0.82	0.20	1.15	1.00	1.41	1.09	1.12	0.68	1.24
Som	1.15	1.70	1.27	1.58	1.11	1.57	1.22	1.30	1.19	1.53	2.70

NDS, nondepressive symptom group. DS, depressive symptom group. The names of the nodes (‘Blame’, ‘FE’, ‘FS’, ‘HL’, ‘HS’, ‘Imp’, ‘Learn’, ‘Lon’, ‘LT’, ‘Pho’, ‘PHQ-9’, ‘Sen’, ‘Soc’, ‘Som’) were ‘Self-blaming’, ‘Family environment’, ‘Friendship’, ‘Hopelessness’, ‘Help-seeking’, ‘Impulsivity’, ‘Learning anxiety’, ‘Loneliness’, ‘Loss of temper’, ‘Phobia’, ‘PHQ-9 score’, ‘Oversensitivity’, ‘Social anxiety’, and ‘Somatic anxiety’. PHQ-9, the 9-item Patient Health Questionnaire

### Network accuracy and stability

The results of the edge weight bootstrap analysis (Fig. S3) presented an overlap among the 95% CIs of the edge weights, especially in the strongest edges. Fig. S4 indicates a high stability of the centrality estimates. The results of the bootstrapped difference tests between edge weights and node centralities are listed in Fig. S5–S6. For the robustness analysis, the CS-coefficients for node strength were above 0.50, and those for closeness were above 0.40 in the two groups from t1 to t4 (Table S11), suggesting that the network was sufficiently stable and that a greater number of cases could be dropped from the original sample without significant changes in the magnitude of the centrality estimates. However, the CS-coefficient for betweenness ranged from 0.13 to 0.28 in the NDS group and ranged from 0.05 to 0.36 in the DS group, which partly reduced the strong support of stability reached by the two other indices and should be interpreted cautiously.

## Discussion

In this longitudinal study, we used a nondiagnostic framework to explore complex associations between DSs and associated factors at the network level in the NDS and DS groups across four time points. Our main findings suggest that the network connection strength varied with symptom severity over time, and unique central nodes were identified for the NDS and DS groups as oversensitivity and impulsivity (3 out of 4 time points), respectively. Additionally, the NDS group exhibited relatively stable connection strengths. These findings are consistent with our hypothesis that adolescents in the NDS group may have experienced some degree of anxiety.

In the global network metrics, the NDS and DS groups showed no significant differences over time. However, the network structure invariance between the two groups differed significantly at t1, and the DS group exhibited larger edge weights between impulsivity and loss of temper than the NDS group. In addition, the central factor with the highest average node strength in the DS group was impulsivity. Prior research suggests that impulsivity moderates the relationship between DSs and risk behaviors during adolescence [43, 44]. In addition, loss of temper and irritable mood are associated with subsequent DSs assessed in follow-ups [18], and in adolescents with depression, irritable mood is identified as a core diagnostic symptom [3]. Moreover, a recent study indicated that irritable mood is a prominent central node in the network analysis of depressive and anxiety symptoms among adolescents [22]. Furthermore, adolescents with impulsivity or irritable mood are prone to use poor emotion regulation strategies in response to negative events, which may contribute to the generation of depressed mood [44, 45].

The current findings revealed that the central factor with the highest node strength in the NDS group over four time points was worry and oversensitivity. Intriguingly, the NDS group presented similar top positive edge weights over time, including those between learning anxiety and social anxiety and those between somatic anxiety and phobia. Previous research indicated that oversensitivity appears to reflect an individual feeling of being overwhelmed by a general sense of worry, which is strongly linked to developmental changes with age and cognitive ability [14, 46]. In addition, worry and oversensitivity may longitudinally predict depression and anxiety disorders, especially in subpopulations of adolescents with high and increasing anxiety levels [14, 47]. Notably, learning anxiety is the most prevalent anxiety subtype in Chinese adolescents [48, 49]. From an early age, Chinese youth need to handle great academic pressure and a substantial amount of homework and face high expectations and strict supervision from their parents [46, 50]. Additionally, prior studies have revealed that high academic stress has been linked to depressive and anxiety symptoms in adolescents [48, 49, 51]. Moreover, social anxiety states appear to be stable in adolescents, and higher social anxiety is associated with poorer psychosocial adjustment [52].

Considering values of node strength from baseline to follow-up, different network patterns emerged in the NDS and DS groups. Additionally, with the NCT, significant differences in node strength were noted in oversensitivity and impulsivity between the NDS and DS groups over time. At the network level, there were no significant findings in the comparisons of node strength between networks in the DS group over time, but the edge weights in the DS group differed. Given the relatively low Jaccard similarity coefficient in the DS group among the four time points, adolescents who reported DSs partly overlapped and varied somewhat over time. Prior research has reported that adolescents experience greater mood instability than adults [53]. Additionally, adolescence is a period with low emotion differentiation [54], and emotion differentiation also fluctuates within individuals over time [55]. Moreover, neuroendocrine changes during puberty affect alterations of brain regions implicated in emotion processing that may relate to the changes in adolescents’ self-reported emotional experiences [56]. Furthermore, contributing factors associated with DSs at the population level may be different and complex over time, which may be linked to the heterogeneity of depression, especially in adolescents [3, 57].

This study has some limitations. First, self-report questionnaires were used, and the screening results may not be sufficient to confirm a clinical diagnosis of depression. Therefore, professional assistance should be sought, and comprehensive evaluations should be performed for those whose PHQ-9 scores indicate possible depressive disorders [58]. Second, while the sample size of this longitudinal study was adequate to perform the analysis, future studies with a larger and more diverse population, may help confirm and extend the present findings. Third, other factors, such as genetic liability, adverse childhood experiences, screen use and family socioeconomic status, may be associated with DSs in adolescents. Future longitudinal research examining DSs in adolescents could benefit from focusing on these research areas to enhance our understanding of the complex and multifactorial nature of depressive symptoms in this population.

## Conclusion

This study found that the network connection strength varied with symptom severity over time, and a higher severity of DSs may have introduced a degree of stability in the network. Depressive severity may have altered the structure of the symptom network and potential risk predictors. These findings have important implications for clinical practice and school educators, particularly when assisting adolescents suffering from depression. Future research and preventive strategies and intervention efforts should consider targeting a reduction if nonclinical DSs to prevent the consolidation of antecedent or risk-symptom relationships.

### Electronic supplementary material

Below is the link to the electronic supplementary material.

Supplementary Material 1

## Data Availability

The authors declare that all data supporting the findings of this study are available within the paper and its supporting information. The data are available from the corresponding author upon reasonable request.
