# Supplementary material for: Using a longitudinal network structure to subgroup depressive symptoms among adolescents
Source: BMC Psychol. 2024 Jan 24;12:46. doi: 10.1186/s40359-024-01537-8 (PMC10807250; doi:10.1186/s40359-024-01537-8)
Supplement: Supplementary file 1 — Supplementary Material 1 [file 40359_2024_1537_MOESM1_ESM.docx]

**Using a Longitudinal Network Structure to Subgroup Depressive Symptoms among Adolescents**

**Supplementary Information**

**Supplementary Methods**

**Supplementary Tables**

Table S1. Comparison of scale scores in each group between baseline and follow-ups

Table S2. Correlation matrix of the network in the NDS group at baseline (t1)

Table S3. Correlation matrix of the network in the DS group at baseline (t1)

Table S4. Correlation matrix of the network in the NDS group at t2

Table S5. Correlation matrix of the network in the DS group at t2

Table S6. Correlation matrix of the network in the NDS group at t3

Table S7. Correlation matrix of the network in the DS group at t3

Table S8. Correlation matrix of the network in the NDS group at t4

Table S9. Correlation matrix of the network in the DS group at t4

Table S10. Comparison of node strength between DS and/or NDS groups

Table S11. Correlation stability coefficient of centrality indices in NDS and DS groups

**Supplementary Figures**

Figure S1 Data screening flowchart

Figure S2. Heatmap of correlation matrix across the entire sample at four time points

Figure S3. Bootstrapped 95% confidence intervals (CI) for the estimated edge weights in the networks

Figure S4. Case-dropping bootstrap for the networks

Figure S5. Bootstrapped difference tests of edge weights in the networks

Figure S6. Bootstrapped difference tests of strength centrality in the networks

**Supplementary Methods**

Baseline data collection took place at the beginning of the spring semester in 2020 (*t1*). The current study had three follow-ups; *t2* at the beginning of the fall semester in 2020, *t3* at the beginning of the spring semester in 2021, and *t4* at the beginning of the fall semester in 2021. Data were fully anonymized in this study.

The survey had six additional questions that were rated on a five-point Likert scale of 1–5 and assessed for family environment, friendship, help-seeking, and loss of temper, hopelessness and lying, respectively. The family environment item stated: "I feel warm and happy at home", and 1 = not at all like my family and 5 = almost always like my family. The friendship item stated: "I have some close and good friends", and 1 = truly false for me and 5 = truly true for me. The help-seeking item stated: "When I encounter problems that are difficult to solve, I will take the initiative to seek help", and 1 = truly false for me and 5 = truly true for me. The loss of temper item stated: "I often want to yell and smash things", and 1 = truly false for me and 5 = truly true for me. The hopelessness item stated: "I feel that my life is full of hope", and 1 = truly true for me and 5 = truly false for me. The lying test was designed to test “lying” and stated: "I have never lied", and 1 = truly true for me and 5 = truly false for me.

**Supplementary Tables**

**Table S1.** **Comparison of scale scores in each group between baseline and follow-ups**

| Comparisons | NDS group | | | |  | DS group | | |
| --- | --- | --- | --- | --- | --- | --- | --- | --- |
|  | Kruskal-Wallis H test | *Post hoc* test | | | Kruskal-Wallis H test | *Post hoc* test | | |
|  |  | t1 v t2 | t1 v t3 | t1 v t4 |  | t1 v t2 | t1 v t3 | t1 v t4 |
| PHQ-9 | 0.93 | 0.63 | 0.79 | 0.73 | 0.36 | 0.68 | 0.23 | 0.91 |
| General MHT | 0.00 | 0.16 | 0.89 | 0.00 | 0.15 | 0.81 | 0.30 | 0.81 |
| Learning anxiety | 0.01 | 0.32 | 0.24 | 0.02 | 0.07 | 0.43 | 0.07 | 0.51 |
| Social anxiety | 0.08 | 0.74 | 0.10 | 0.39 | 0.33 | 0.82 | 0.09 | 0.52 |
| Loneliness | 0.00 | 0.12 | 0.13 | 0.00 | 0.34 | 0.38 | 0.42 | 0.65 |
| Self-blaming | 0.01 | 0.08 | 0.79 | 0.00 | 0.15 | 0.44 | 0.29 | 0.36 |
| Oversensitivity | 0.00 | 0.14 | 0.06 | 0.16 | 0.00 | 0.90 | 0.00 | 0.08 |
| Somatic anxiety | 0.01 | 0.44 | 0.83 | 0.00 | 0.02 | 0.42 | 0.02 | 0.30 |
| Phobia anxiety | 0.00 | 0.67 | 0.00 | 0.00 | 0.00 | 0.65 | 0.00 | 0.00 |
| Impulsivity | 0.04 | 0.11 | 0.11 | 0.00 | 0.21 | 0.48 | 0.52 | 0.20 |

p values for Kruskal-Wallis H test and *post hoc* tests. NDS, nondepressive symptom group. DS, depressive symptom group. PHQ-9, the 9-item Patient Health Questionnaire. MHT, the Mental Health Test.

**Table S2. Correlation matrix of the network in the NDS group at baseline (t1)**

|  | Learn | Soc | Lon | Blame | Sen | Som | Pho | Imp | FE | FS | LT | HL | HS | PHQ-9 |
| --- | --- | --- | --- | --- | --- | --- | --- | --- | --- | --- | --- | --- | --- | --- |
| Learn |  | **0.35** | 0.06 | 0.18 | 0.08 | 0.10 | 0.04 | 0.00 | 0.00 | 0.00 | 0.03 | 0.02 | -0.01 | 0.04 |
| Soc | **0.35** |  | 0.10 | 0.13 | 0.17 | 0.11 | 0.04 | 0.02 | -0.01 | 0.00 | 0.02 | 0.00 | 0.00 | 0.07 |
| Lon | 0.06 | 0.10 |  | 0.09 | 0.05 | 0.11 | 0.00 | 0.06 | -0.01 | -0.19 | 0.07 | 0.00 | -0.06 | 0.00 |
| Blame | 0.18 | 0.13 | 0.09 |  | **0.31** | 0.08 | 0.04 | 0.00 | 0.00 | 0.00 | 0.00 | 0.01 | 0.00 | 0.00 |
| Sen | 0.08 | 0.17 | 0.05 | **0.31** |  | 0.21 | 0.11 | 0.19 | 0.00 | 0.00 | 0.00 | 0.00 | 0.00 | 0.03 |
| Som | 0.10 | 0.11 | 0.11 | 0.08 | 0.21 |  | **0.23** | 0.13 | 0.00 | 0.00 | 0.00 | 0.03 | 0.00 | 0.04 |
| Pho | 0.04 | 0.04 | 0.00 | 0.04 | 0.11 | **0.23** |  | 0.16 | 0.00 | 0.00 | 0.00 | 0.00 | 0.00 | 0.08 |
| Imp | 0.00 | 0.02 | 0.06 | 0.00 | 0.19 | 0.13 | 0.16 |  | -0.06 | 0.00 | 0.12 | 0.04 | -0.08 | 0.05 |
| FE | 0.00 | -0.01 | -0.01 | 0.00 | 0.00 | 0.00 | 0.00 | -0.06 |  | 0.20 | -0.04 | -0.26 | 0.00 | -0.09 |
| FS | 0.00 | 0.00 | -0.19 | 0.00 | 0.00 | 0.00 | 0.00 | 0.00 | 0.20 |  | 0.00 | -0.10 | 0.17 | 0.00 |
| LT | 0.03 | 0.02 | 0.07 | 0.00 | 0.00 | 0.00 | 0.00 | 0.12 | -0.04 | 0.00 |  | 0.00 | 0.00 | 0.07 |
| HL | 0.02 | 0.00 | 0.00 | 0.01 | 0.00 | 0.03 | 0.00 | 0.04 | -0.26 | -0.10 | 0.00 |  | -0.28 | 0.13 |
| HS | -0.01 | 0.00 | -0.06 | 0.00 | 0.00 | 0.00 | 0.00 | -0.08 | 0.00 | 0.17 | 0.00 | -0.28 |  | -0.08 |
| PHQ-9 | 0.04 | 0.07 | 0.00 | 0.00 | 0.03 | 0.04 | 0.08 | 0.05 | -0.09 | 0.00 | 0.07 | 0.13 | -0.08 |  |

The names of the nodes ('Learn', 'Soc', 'Lon', 'Blame', 'Sen', 'Som', 'Pho', 'Imp', 'FE', 'FS', 'LT', 'HL', 'HS' and 'PHQ-9') were 'Learning anxiety', 'Social anxiety', 'Loneliness', 'Self-blaming', 'Oversensitivity', 'Somatic anxiety', 'Phobia', 'Impulsivity', 'Family environment', 'Friendship', 'Loss of temper', 'Hopelessness', 'Help-seeking' and 'PHQ-9 score'. PHQ-9, the 9-item Patient Health Questionnaire. The top three positive edge weights list in bold font.

**Table S3. Correlation matrix of the network in the DS group at baseline (t1)**

|  | Learn | Soc | Lon | Blame | Sen | Som | Pho | Imp | FE | FS | LT | HL | HS | PHQ-9 |
| --- | --- | --- | --- | --- | --- | --- | --- | --- | --- | --- | --- | --- | --- | --- |
| Learn |  | 0.21 | 0.00 | 0.24 | 0.10 | 0.21 | 0.00 | 0.00 | 0.00 | 0.02 | 0.00 | 0.00 | 0.00 | 0.03 |
| Soc | 0.21 |  | 0.14 | 0.20 | 0.11 | 0.09 | 0.00 | 0.11 | 0.00 | 0.00 | 0.00 | 0.00 | 0.00 | 0.05 |
| Lon | 0.00 | 0.14 |  | 0.12 | 0.11 | 0.09 | 0.06 | 0.02 | -0.04 | -0.22 | 0.00 | 0.09 | 0.00 | 0.04 |
| Blame | 0.24 | 0.20 | 0.12 |  | 0.13 | 0.02 | 0.09 | 0.00 | 0.00 | 0.00 | 0.00 | 0.00 | 0.00 | 0.00 |
| Sen | 0.10 | 0.11 | 0.11 | 0.13 |  | 0.23 | 0.14 | 0.08 | 0.00 | -0.03 | 0.00 | 0.00 | 0.00 | 0.00 |
| Som | 0.21 | 0.09 | 0.09 | 0.02 | 0.23 |  | 0.24 | **0.25** | 0.00 | 0.00 | 0.00 | 0.00 | -0.01 | 0.05 |
| Pho | 0.00 | 0.00 | 0.06 | 0.09 | 0.14 | 0.24 |  | 0.13 | 0.00 | 0.00 | 0.00 | 0.00 | 0.00 | 0.00 |
| Imp | 0.00 | 0.11 | 0.02 | 0.00 | 0.08 | **0.25** | 0.13 |  | -0.07 | 0.00 | **0.33** | 0.07 | -0.01 | 0.13 |
| FE | 0.00 | 0.00 | -0.04 | 0.00 | 0.00 | 0.00 | 0.00 | -0.07 |  | 0.10 | -0.01 | -0.22 | 0.16 | -0.05 |
| FS | 0.02 | 0.00 | -0.22 | 0.00 | -0.03 | 0.00 | 0.00 | 0.00 | 0.10 |  | 0.00 | -0.11 | 0.05 | 0.00 |
| LT | 0.00 | 0.00 | 0.00 | 0.00 | 0.00 | 0.00 | 0.00 | **0.33** | -0.01 | 0.00 |  | 0.02 | -0.09 | 0.06 |
| HL | 0.00 | 0.00 | 0.09 | 0.00 | 0.00 | 0.00 | 0.00 | 0.07 | -0.22 | -0.11 | 0.02 |  | -0.16 | **0.30** |
| HS | 0.00 | 0.00 | 0.00 | 0.00 | 0.00 | -0.01 | 0.00 | -0.01 | 0.16 | 0.05 | -0.09 | -0.16 |  | -0.05 |
| PHQ-9 | 0.03 | 0.05 | 0.04 | 0.00 | 0.00 | 0.05 | 0.00 | 0.13 | -0.05 | 0.00 | 0.06 | **0.30** | -0.05 |  |

The names of the nodes ('Learn', 'Soc', 'Lon', 'Blame', 'Sen', 'Som', 'Pho', 'Imp', 'FE', 'FS', 'LT', 'HL', 'HS' and 'PHQ-9') were 'Learning anxiety', 'Social anxiety', 'Loneliness', 'Self-blaming', 'Oversensitivity', 'Somatic anxiety', 'Phobia', 'Impulsivity', 'Family environment', 'Friendship', 'Loss of temper', 'Hopelessness', 'Help-seeking' and 'PHQ-9 score'. PHQ-9, the 9-item Patient Health Questionnaire. The top three positive edge weights list in bold font.

**Table S4. Correlation matrix of the network in the NDS group at t2**

|  | Learn | Soc | Lon | Blame | Sen | Som | Pho | Imp | FE | FS | LT | HL | HS | PHQ-9 |
| --- | --- | --- | --- | --- | --- | --- | --- | --- | --- | --- | --- | --- | --- | --- |
| Learn |  | **0.32** | 0.01 | 0.21 | 0.17 | 0.06 | 0.09 | 0.00 | 0.00 | 0.00 | 0.00 | 0.02 | -0.01 | 0.07 |
| Soc | **0.32** |  | 0.12 | 0.17 | 0.18 | 0.14 | 0.00 | 0.01 | -0.01 | 0.00 | 0.00 | 0.01 | 0.00 | 0.01 |
| Lon | 0.01 | 0.12 |  | 0.02 | 0.07 | 0.07 | 0.02 | 0.08 | 0.00 | -0.17 | 0.00 | 0.08 | -0.07 | 0.00 |
| Blame | **0.21** | 0.17 | 0.02 |  | **0.21** | 0.12 | 0.02 | 0.00 | 0.00 | 0.00 | 0.00 | 0.00 | 0.00 | 0.00 |
| Sen | 0.17 | 0.18 | 0.07 | 0.21 |  | 0.16 | 0.17 | 0.20 | 0.00 | 0.00 | 0.00 | 0.00 | -0.01 | 0.10 |
| Som | 0.06 | 0.14 | 0.07 | 0.12 | 0.16 |  | **0.22** | 0.20 | 0.00 | 0.00 | 0.05 | 0.00 | 0.00 | 0.08 |
| Pho | 0.09 | 0.00 | 0.02 | 0.02 | 0.17 | **0.22** |  | 0.11 | 0.00 | 0.01 | 0.02 | 0.00 | 0.00 | 0.04 |
| Imp | 0.00 | 0.01 | 0.08 | 0.00 | 0.20 | 0.20 | 0.11 |  | 0.00 | 0.00 | 0.15 | 0.07 | -0.01 | 0.00 |
| FE | 0.00 | -0.01 | 0.00 | 0.00 | 0.00 | 0.00 | 0.00 | 0.00 |  | 0.15 | -0.08 | -0.21 | 0.06 | -0.11 |
| FS | 0.00 | 0.00 | -0.17 | 0.00 | 0.00 | 0.00 | 0.01 | 0.00 | 0.15 |  | 0.00 | -0.11 | 0.12 | 0.00 |
| LT | 0.00 | 0.00 | 0.00 | 0.00 | 0.00 | 0.05 | 0.02 | 0.15 | -0.08 | 0.00 |  | 0.03 | 0.00 | 0.02 |
| HL | 0.02 | 0.01 | 0.08 | 0.00 | 0.00 | 0.00 | 0.00 | 0.07 | -0.21 | -0.11 | 0.03 |  | -0.25 | 0.17 |
| HS | -0.01 | 0.00 | -0.07 | 0.00 | -0.01 | 0.00 | 0.00 | -0.01 | 0.06 | 0.12 | 0.00 | -0.25 |  | -0.12 |
| PHQ-9 | 0.07 | 0.01 | 0.00 | 0.00 | 0.10 | 0.08 | 0.04 | 0.00 | -0.11 | 0.00 | 0.02 | 0.17 | -0.12 |  |

The names of the nodes ('Learn', 'Soc', 'Lon', 'Blame', 'Sen', 'Som', 'Pho', 'Imp', 'FE', 'FS', 'LT', 'HL', 'HS' and 'PHQ-9') were 'Learning anxiety', 'Social anxiety', 'Loneliness', 'Self-blaming', 'Oversensitivity', 'Somatic anxiety', 'Phobia', 'Impulsivity', 'Family environment', 'Friendship', 'Loss of temper', 'Hopelessness', 'Help-seeking' and 'PHQ-9 score'. PHQ-9, the 9-item Patient Health Questionnaire. The top three positive edge weights list in bold font.

**Table S5. Correlation matrix of the network in the DS group at t2**

|  | Learn | Soc | Lon | Blame | Sen | Som | Pho | Imp | FE | FS | LT | HL | HS | PHQ-9 |
| --- | --- | --- | --- | --- | --- | --- | --- | --- | --- | --- | --- | --- | --- | --- |
| Learn |  | **0.30** | 0.00 | **0.32** | 0.08 | 0.07 | 0.03 | 0.00 | 0.00 | 0.08 | 0.05 | 0.00 | 0.01 | 0.00 |
| Soc | **0.30** |  | 0.13 | 0.02 | 0.10 | 0.10 | 0.03 | 0.07 | 0.00 | -0.02 | 0.00 | 0.00 | -0.06 | 0.09 |
| Lon | 0.00 | 0.13 |  | 0.07 | 0.00 | 0.13 | 0.01 | 0.04 | -0.01 | -0.32 | 0.00 | 0.11 | -0.05 | 0.07 |
| Blame | **0.32** | 0.02 | 0.07 |  | 0.21 | 0.17 | 0.08 | 0.00 | 0.00 | 0.00 | 0.00 | 0.00 | 0.00 | 0.00 |
| Sen | 0.08 | 0.10 | 0.00 | 0.21 |  | 0.22 | 0.20 | 0.15 | 0.00 | 0.00 | 0.00 | 0.00 | 0.00 | 0.00 |
| Som | 0.07 | 0.10 | 0.13 | 0.17 | 0.22 |  | 0.14 | **0.21** | 0.00 | 0.00 | 0.00 | 0.00 | -0.03 | 0.12 |
| Pho | 0.03 | 0.03 | 0.01 | 0.08 | 0.20 | 0.14 |  | 0.18 | 0.06 | 0.00 | 0.00 | 0.00 | 0.00 | 0.00 |
| Imp | 0.00 | 0.07 | 0.04 | 0.00 | 0.15 | **0.21** | 0.18 |  | -0.06 | 0.00 | 0.28 | 0.05 | 0.00 | 0.17 |
| FE | 0.00 | 0.00 | -0.01 | 0.00 | 0.00 | 0.00 | 0.06 | -0.06 |  | 0.11 | -0.05 | -0.29 | 0.04 | -0.12 |
| FS | 0.08 | -0.02 | -0.32 | 0.00 | 0.00 | 0.00 | 0.00 | 0.00 | 0.11 |  | 0.07 | -0.11 | 0.12 | 0.00 |
| LT | 0.05 | 0.00 | 0.00 | 0.00 | 0.00 | 0.00 | 0.00 | 0.28 | -0.05 | 0.07 |  | 0.00 | 0.00 | 0.06 |
| HL | 0.00 | 0.00 | 0.11 | 0.00 | 0.00 | 0.00 | 0.00 | 0.05 | -0.29 | -0.11 | 0.00 |  | -0.19 | 0.18 |
| HS | 0.01 | -0.06 | -0.05 | 0.00 | 0.00 | -0.03 | 0.00 | 0.00 | 0.04 | 0.12 | 0.00 | -0.19 |  | -0.09 |
| PHQ-9 | 0.00 | 0.09 | 0.07 | 0.00 | 0.00 | 0.12 | 0.00 | 0.17 | -0.12 | 0.00 | 0.06 | 0.18 | -0.09 |  |

The names of the nodes ('Learn', 'Soc', 'Lon', 'Blame', 'Sen', 'Som', 'Pho', 'Imp', 'FE', 'FS', 'LT', 'HL', 'HS' and 'PHQ-9') were 'Learning anxiety', 'Social anxiety', 'Loneliness', 'Self-blaming', 'Oversensitivity', 'Somatic anxiety', 'Phobia', 'Impulsivity', 'Family environment', 'Friendship', 'Loss of temper', 'Hopelessness', 'Help-seeking' and 'PHQ-9 score'. PHQ-9, the 9-item Patient Health Questionnaire. The top three positive edge weights list in bold font.

**Table S6. Correlation matrix of the network in the NDS group at t3**

|  | Learn | Soc | Lon | Blame | Sen | Som | Pho | Imp | FE | FS | LT | HL | HS | PHQ-9 |
| --- | --- | --- | --- | --- | --- | --- | --- | --- | --- | --- | --- | --- | --- | --- |
| Learn |  | **0.32** | 0.01 | 0.16 | 0.13 | 0.13 | 0.01 | 0.00 | 0.00 | 0.00 | 0.02 | 0.00 | 0.00 | 0.05 |
| Soc | **0.32** |  | 0.08 | 0.12 | 0.21 | 0.13 | 0.03 | 0.06 | 0.00 | 0.00 | 0.00 | 0.05 | -0.02 | 0.03 |
| Lon | 0.01 | 0.08 |  | 0.09 | 0.08 | 0.06 | 0.04 | 0.05 | 0.00 | -0.14 | 0.00 | 0.00 | -0.07 | 0.01 |
| Blame | 0.16 | 0.12 | 0.09 |  | **0.24** | 0.11 | 0.00 | 0.00 | 0.00 | 0.00 | 0.00 | 0.00 | 0.00 | 0.06 |
| Sen | 0.13 | 0.21 | 0.08 | **0.24** |  | **0.18** | 0.15 | 0.19 | 0.00 | 0.00 | 0.00 | 0.00 | 0.00 | 0.10 |
| Som | 0.13 | 0.13 | 0.06 | 0.11 | **0.18** |  | 0.22 | 0.14 | 0.00 | 0.00 | 0.05 | 0.00 | 0.00 | 0.02 |
| Pho | 0.01 | 0.03 | 0.04 | 0.00 | 0.15 | 0.22 |  | 0.12 | 0.00 | 0.00 | 0.00 | 0.00 | 0.00 | 0.00 |
| Imp | 0.00 | 0.06 | 0.05 | 0.00 | 0.19 | 0.14 | 0.12 |  | -0.05 | 0.00 | 0.13 | 0.06 | 0.00 | 0.08 |
| FE | 0.00 | 0.00 | 0.00 | 0.00 | 0.00 | 0.00 | 0.00 | -0.05 |  | 0.12 | 0.00 | -0.20 | 0.04 | -0.13 |
| FS | 0.00 | 0.00 | -0.14 | 0.00 | 0.00 | 0.00 | 0.00 | 0.00 | 0.12 |  | 0.00 | -0.12 | 0.08 | -0.02 |
| LT | 0.02 | 0.00 | 0.00 | 0.00 | 0.00 | 0.05 | 0.00 | 0.13 | 0.00 | 0.00 |  | 0.01 | 0.00 | 0.07 |
| HL | 0.00 | 0.05 | 0.00 | 0.00 | 0.00 | 0.00 | 0.00 | 0.06 | -0.20 | -0.12 | 0.01 |  | -0.14 | 0.15 |
| HS | 0.00 | -0.02 | -0.07 | 0.00 | 0.00 | 0.00 | 0.00 | 0.00 | 0.04 | 0.08 | 0.00 | -0.14 |  | -0.16 |
| PHQ-9 | 0.05 | 0.03 | 0.01 | 0.06 | 0.10 | 0.02 | 0.00 | 0.08 | -0.13 | -0.02 | 0.07 | 0.15 | -0.16 |  |

The names of the nodes ('Learn', 'Soc', 'Lon', 'Blame', 'Sen', 'Som', 'Pho', 'Imp', 'FE', 'FS', 'LT', 'HL', 'HS' and 'PHQ-9') were 'Learning anxiety', 'Social anxiety', 'Loneliness', 'Self-blaming', 'Oversensitivity', 'Somatic anxiety', 'Phobia', 'Impulsivity', 'Family environment', 'Friendship', 'Loss of temper', 'Hopelessness', 'Help-seeking' and 'PHQ-9 score'. PHQ-9, the 9-item Patient Health Questionnaire. The top three positive edge weights list in bold font.

**Table S7. Correlation matrix of the network in the DS group at t3**

|  | Learn | Soc | Lon | Blame | Sen | Som | Pho | Imp | FE | FS | LT | HL | HS | PHQ-9 |
| --- | --- | --- | --- | --- | --- | --- | --- | --- | --- | --- | --- | --- | --- | --- |
| Learn |  | 0.24 | 0.00 | 0.24 | 0.19 | 0.04 | 0.03 | 0.00 | 0.00 | 0.00 | 0.00 | 0.00 | 0.00 | 0.01 |
| Soc | 0.24 |  | 0.12 | 0.20 | 0.12 | 0.12 | 0.08 | 0.08 | 0.00 | 0.00 | 0.07 | 0.00 | 0.00 | 0.01 |
| Lon | 0.00 | 0.12 |  | 0.11 | 0.00 | 0.10 | 0.00 | 0.05 | 0.00 | -0.22 | 0.00 | 0.04 | -0.14 | 0.09 |
| Blame | 0.24 | 0.20 | 0.11 |  | 0.09 | 0.03 | 0.11 | 0.02 | 0.00 | 0.00 | 0.00 | 0.00 | 0.00 | 0.02 |
| Sen | 0.19 | 0.12 | 0.00 | 0.09 |  | 0.23 | 0.13 | 0.15 | 0.00 | 0.00 | 0.00 | 0.00 | 0.00 | 0.00 |
| Som | 0.04 | 0.12 | 0.10 | 0.03 | 0.23 |  | 0.23 | **0.28** | -0.03 | 0.00 | 0.00 | 0.00 | -0.03 | 0.11 |
| Pho | 0.03 | 0.08 | 0.00 | 0.11 | 0.13 | 0.23 |  | 0.03 | -0.01 | 0.00 | 0.00 | 0.00 | 0.00 | 0.00 |
| Imp | 0.00 | 0.08 | 0.05 | 0.02 | 0.15 | **0.28** | 0.03 |  | -0.04 | 0.00 | **0.26** | 0.09 | 0.00 | 0.14 |
| FE | 0.00 | 0.00 | 0.00 | 0.00 | 0.00 | -0.03 | -0.01 | -0.04 |  | 0.14 | -0.03 | -0.29 | 0.00 | -0.02 |
| FS | 0.00 | 0.00 | -0.22 | 0.00 | 0.00 | 0.00 | 0.00 | 0.00 | 0.14 |  | 0.00 | -0.08 | 0.08 | 0.00 |
| LT | 0.00 | 0.07 | 0.00 | 0.00 | 0.00 | 0.00 | 0.00 | **0.26** | -0.03 | 0.00 |  | 0.08 | 0.00 | 0.07 |
| HL | 0.00 | 0.00 | 0.04 | 0.00 | 0.00 | 0.00 | 0.00 | 0.09 | -0.29 | -0.08 | 0.08 |  | -0.26 | **0.28** |
| HS | 0.00 | 0.00 | -0.14 | 0.00 | 0.00 | -0.03 | 0.00 | 0.00 | 0.00 | 0.08 | 0.00 | -0.26 |  | -0.03 |
| PHQ-9 | 0.01 | 0.01 | 0.09 | 0.02 | 0.00 | 0.11 | 0.00 | 0.14 | -0.02 | 0.00 | 0.07 | **0.28** | -0.03 |  |

The names of the nodes ('Learn', 'Soc', 'Lon', 'Blame', 'Sen', 'Som', 'Pho', 'Imp', 'FE', 'FS', 'LT', 'HL', 'HS' and 'PHQ-9') were 'Learning anxiety', 'Social anxiety', 'Loneliness', 'Self-blaming', 'Oversensitivity', 'Somatic anxiety', 'Phobia', 'Impulsivity', 'Family environment', 'Friendship', 'Loss of temper', 'Hopelessness', 'Help-seeking' and 'PHQ-9 score'. PHQ-9, the 9-item Patient Health Questionnaire. The top three positive edge weights list in bold font.

**Table S8. Correlation matrix of the network in the NDS group at t4**

|  | Learn | Soc | Lon | Blame | Sen | Som | Pho | Imp | FE | FS | LT | HL | HS | PHQ-9 |
| --- | --- | --- | --- | --- | --- | --- | --- | --- | --- | --- | --- | --- | --- | --- |
| Learn |  | **0.31** | 0.01 | 0.22 | 0.16 | 0.10 | 0.03 | 0.00 | 0.00 | 0.00 | 0.00 | 0.00 | 0.00 | 0.06 |
| Soc | **0.31** |  | 0.13 | **0.24** | 0.13 | 0.17 | 0.02 | 0.12 | 0.00 | 0.00 | 0.00 | 0.00 | 0.00 | 0.00 |
| Lon | 0.01 | 0.13 |  | 0.05 | 0.07 | 0.05 | 0.02 | 0.05 | 0.00 | -0.18 | 0.00 | 0.03 | -0.12 | 0.00 |
| Blame | 0.22 | **0.24** | 0.05 |  | 0.22 | 0.05 | 0.02 | 0.00 | 0.00 | 0.00 | 0.00 | 0.00 | 0.00 | 0.00 |
| Sen | 0.16 | 0.13 | 0.07 | 0.22 |  | 0.18 | 0.16 | 0.13 | 0.00 | 0.00 | 0.00 | 0.00 | -0.03 | 0.08 |
| Som | 0.10 | 0.17 | 0.05 | 0.05 | 0.18 |  | **0.27** | 0.17 | 0.00 | 0.00 | 0.00 | 0.00 | 0.00 | 0.09 |
| Pho | 0.03 | 0.02 | 0.02 | 0.02 | 0.16 | **0.27** |  | 0.04 | 0.00 | 0.00 | 0.04 | 0.00 | 0.00 | 0.00 |
| Imp | 0.00 | 0.12 | 0.05 | 0.00 | 0.13 | 0.17 | 0.04 |  | -0.10 | 0.00 | 0.16 | 0.05 | -0.02 | 0.07 |
| FE | 0.00 | 0.00 | 0.00 | 0.00 | 0.00 | 0.00 | 0.00 | -0.10 |  | 0.15 | -0.03 | -0.27 | 0.03 | -0.16 |
| FS | 0.00 | 0.00 | -0.18 | 0.00 | 0.00 | 0.00 | 0.00 | 0.00 | 0.15 |  | 0.00 | -0.11 | 0.08 | -0.01 |
| LT | 0.00 | 0.00 | 0.00 | 0.00 | 0.00 | 0.00 | 0.04 | 0.16 | -0.03 | 0.00 |  | 0.00 | -0.02 | 0.08 |
| HL | 0.00 | 0.00 | 0.03 | 0.00 | 0.00 | 0.00 | 0.00 | 0.05 | -0.27 | -0.11 | 0.00 |  | -0.21 | 0.14 |
| HS | 0.00 | 0.00 | -0.12 | 0.00 | -0.03 | 0.00 | 0.00 | -0.02 | 0.03 | 0.08 | -0.02 | -0.21 |  | -0.09 |
| PHQ-9 | 0.06 | 0.00 | 0.00 | 0.00 | 0.08 | 0.09 | 0.00 | 0.07 | -0.16 | -0.01 | 0.08 | 0.14 | -0.09 |  |

The names of the nodes ('Learn', 'Soc', 'Lon', 'Blame', 'Sen', 'Som', 'Pho', 'Imp', 'FE', 'FS', 'LT', 'HL', 'HS' and 'PHQ-9') were 'Learning anxiety', 'Social anxiety', 'Loneliness', 'Self-blaming', 'Oversensitivity', 'Somatic anxiety', 'Phobia', 'Impulsivity', 'Family environment', 'Friendship', 'Loss of temper', 'Hopelessness', 'Help-seeking' and 'PHQ-9 score'. PHQ-9, the 9-item Patient Health Questionnaire. The top three positive edge weights list in bold font.

**Table S9. Correlation matrix of the network in the DS group at t4**

|  | Learn | Soc | Lon | Blame | Sen | Som | Pho | Imp | FE | FS | LT | HL | HS | PHQ-9 |
| --- | --- | --- | --- | --- | --- | --- | --- | --- | --- | --- | --- | --- | --- | --- |
| Learn |  | **0.27** | 0.00 | 0.18 | 0.21 | 0.02 | 0.01 | 0.00 | 0.00 | 0.00 | 0.06 | 0.00 | 0.00 | 0.00 |
| Soc | **0.27** |  | 0.09 | 0.23 | 0.06 | 0.19 | 0.14 | 0.04 | 0.00 | 0.00 | 0.00 | 0.02 | -0.01 | 0.03 |
| Lon | 0.00 | 0.09 |  | 0.10 | 0.00 | 0.10 | 0.08 | 0.05 | 0.00 | -0.23 | 0.00 | 0.07 | -0.14 | 0.01 |
| Blame | 0.18 | 0.23 | 0.10 |  | 0.15 | 0.04 | 0.13 | 0.00 | 0.00 | 0.00 | 0.00 | 0.00 | 0.00 | 0.01 |
| Sen | 0.21 | 0.06 | 0.00 | 0.15 |  | 0.19 | 0.06 | 0.20 | 0.00 | 0.04 | 0.00 | 0.00 | 0.00 | 0.00 |
| Som | 0.02 | 0.19 | 0.10 | 0.04 | 0.19 |  | 0.16 | **0.27** | 0.00 | 0.00 | 0.01 | 0.00 | 0.00 | 0.13 |
| Pho | 0.01 | 0.14 | 0.08 | 0.13 | 0.06 | 0.16 |  | 0.15 | 0.00 | 0.00 | 0.00 | 0.00 | 0.00 | 0.00 |
| Imp | 0.00 | 0.04 | 0.05 | 0.00 | 0.20 | **0.27** | 0.15 |  | -0.08 | 0.00 | 0.23 | 0.04 | -0.06 | 0.12 |
| FE | 0.00 | 0.00 | 0.00 | 0.00 | 0.00 | 0.00 | 0.00 | -0.08 |  | 0.16 | -0.01 | -0.32 | 0.06 | -0.07 |
| FS | 0.00 | 0.00 | -0.23 | 0.00 | 0.04 | 0.00 | 0.00 | 0.00 | 0.16 |  | 0.00 | -0.10 | 0.08 | 0.00 |
| LT | 0.06 | 0.00 | 0.00 | 0.00 | 0.00 | 0.01 | 0.00 | 0.23 | -0.01 | 0.00 |  | 0.00 | 0.00 | 0.16 |
| HL | 0.00 | 0.02 | 0.07 | 0.00 | 0.00 | 0.00 | 0.00 | 0.04 | -0.32 | -0.10 | 0.00 |  | -0.19 | **0.25** |
| HS | 0.00 | -0.01 | -0.14 | 0.00 | 0.00 | 0.00 | 0.00 | -0.06 | 0.06 | 0.08 | 0.00 | -0.19 |  | -0.04 |
| PHQ-9 | 0.00 | 0.03 | 0.01 | 0.01 | 0.00 | 0.13 | 0.00 | 0.12 | -0.07 | 0.00 | 0.16 | **0.25** | -0.04 |  |

The names of the nodes ('Learn', 'Soc', 'Lon', 'Blame', 'Sen', 'Som', 'Pho', 'Imp', 'FE', 'FS', 'LT', 'HL', 'HS' and 'PHQ-9') were 'Learning anxiety', 'Social anxiety', 'Loneliness', 'Self-blaming', 'Oversensitivity', 'Somatic anxiety', 'Phobia', 'Impulsivity', 'Family environment', 'Friendship', 'Loss of temper', 'Hopelessness', 'Help-seeking' and 'PHQ-9 score'. PHQ-9, the 9-item Patient Health Questionnaire. The top three positive edge weights list in bold font.

**Table S10. Comparison of node strength between DS and/or NDS groups**

| Networks | Learn | Soc | Lon | Blame | Sen | Som | Pho | Imp | FE | FS | LT | HL | HS | PHQ-9 |
| --- | --- | --- | --- | --- | --- | --- | --- | --- | --- | --- | --- | --- | --- | --- |
| NDS-t1 v DS-t1 | 0.34 | 0.26 | 0.21 | 0.56 | 0.02 | 0.12 | 0.62 | 0.01 | 0.81 | 0.28 | 0.13 | 0.38 | 0.25 | 0.51 |
| NDS-t2 v DS-t2 | 0.93 | 0.36 | 0.03 | 0.15 | 0.00 | 0.40 | 0.58 | 0.00 | 0.34 | 0.08 | 0.18 | 0.84 | 0.57 | 0.07 |
| NDS-t3 v DS-t3 | 0.34 | 0.96 | 0.01 | 0.71 | 0.00 | 0.09 | 0.42 | 0.00 | 0.82 | 0.72 | 0.02 | 0.00 | 0.78 | 0.35 |
| NDS-t4 v DS-t4 | 0.08 | 0.47 | 0.09 | 0.60 | 0.00 | 0.72 | 0.13 | 0.00 | 0.68 | 0.51 | 0.15 | 0.11 | 0.91 | 0.76 |
| DS-t2 v DS-t1 | 0.32 | 0.95 | 0.91 | 0.42 | 0.75 | 0.97 | 0.38 | 0.75 | 0.47 | 0.10 | 1.00 | 0.77 | 0.71 | 0.17 |
| DS-t3 v DS-t1 | 0.61 | 0.13 | 0.64 | 0.69 | 0.93 | 0.90 | 0.81 | 0.66 | 0.57 | 0.98 | 0.99 | 0.25 | 0.97 | 0.93 |
| DS-t4 v DS-t1 | 0.57 | 0.14 | 0.63 | 0.61 | 0.92 | 0.39 | 0.40 | 0.72 | 0.70 | 0.50 | 0.64 | 0.98 | 0.85 | 0.60 |
| NDS-t2 v NDS-t1 | 0.40 | 0.67 | 0.32 | 0.16 | 0.08 | 0.40 | 0.93 | 0.30 | 0.52 | 0.30 | 0.96 | 0.34 | 0.77 | 0.65 |
| NDS-t3 v NDS-t1 | 0.51 | 0.70 | 0.05 | 0.47 | 0.08 | 0.92 | 0.04 | 0.56 | 0.21 | 0.09 | 0.43 | 0.19 | 0.04 | 0.03 |
| NDS-t4 v NDS-t1 | 0.93 | 0.18 | 0.27 | 0.45 | 0.65 | 0.58 | 0.16 | 0.91 | 0.46 | 0.21 | 0.89 | 0.48 | 0.29 | 0.24 |

Results of permutation test are presented in p value (uncorrected). NDS, nondepressive symptom group. DS, depressive symptom group. The scores of node strength between the NDS and DS groups differed significantly for oversensitivity and impulsivity at four time points and for loss of temper, helplessness and loneliness at t3. The values of node strength for phobia, help-seeking behavior and PHQ-9 score were significantly different between t3 and t1 in the NDS group. No other significant differences in node strength were observed between baseline and follow-up in each group.

The names of the nodes ('Learn', 'Soc', 'Lon', 'Blame', 'Sen', 'Som', 'Pho', 'Imp', 'FE', 'FS', 'LT', 'HL', 'HS' and 'PHQ-9') were 'Learning anxiety', 'Social anxiety', 'Loneliness', 'Self-blaming', 'Oversensitivity', 'Somatic anxiety', 'Phobia', 'Impulsivity', 'Family environment', 'Friendship', 'Loss of temper', 'Hopelessness', 'Help-seeking' and 'PHQ-9 score'. PHQ-9, the 9-item Patient Health Questionnaire.

**Table S11. Correlation stability coefficient of centrality indices in NDS and DS groups**

| Centralities | Baseline (t1) | | t2 | | t3 | | t4 | |
| --- | --- | --- | --- | --- | --- | --- | --- | --- |
|  | NDS | DS | NDS | DS | NDS | DS | NDS | DS |
| Strength | 0.75 | 0.59 | 0.75 | 0.67 | 0.75 | 0.67 | 0.75 | 0.67 |
| Closeness | 0.44 | 0.44 | 0.52 | 0.44 | 0.59 | 0.44 | 0.59 | 0.44 |
| Betweenness | 0.21 | 0.36 | 0.13 | 0.13 | 0.21 | 0.05 | 0.28 | 0.13 |

Values are correlation coefficients. The CS coefficient 0.7 represents the maximum proportion of observations that can be dropped such that the correlation between the original centrality metric and those of the sampled subsets is 0.7 or higher with 95% probability. NDS, nondepressive symptom group. DS, depressive symptom group.

**Supplementary Figures**

**
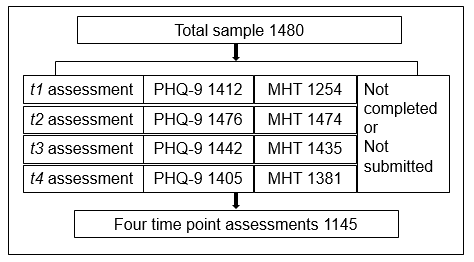
**

**Figure S1 Data screening flowchart**

PHQ-9, 9-item Patient Health Questionnaire. MHT, the Mental Health Test.


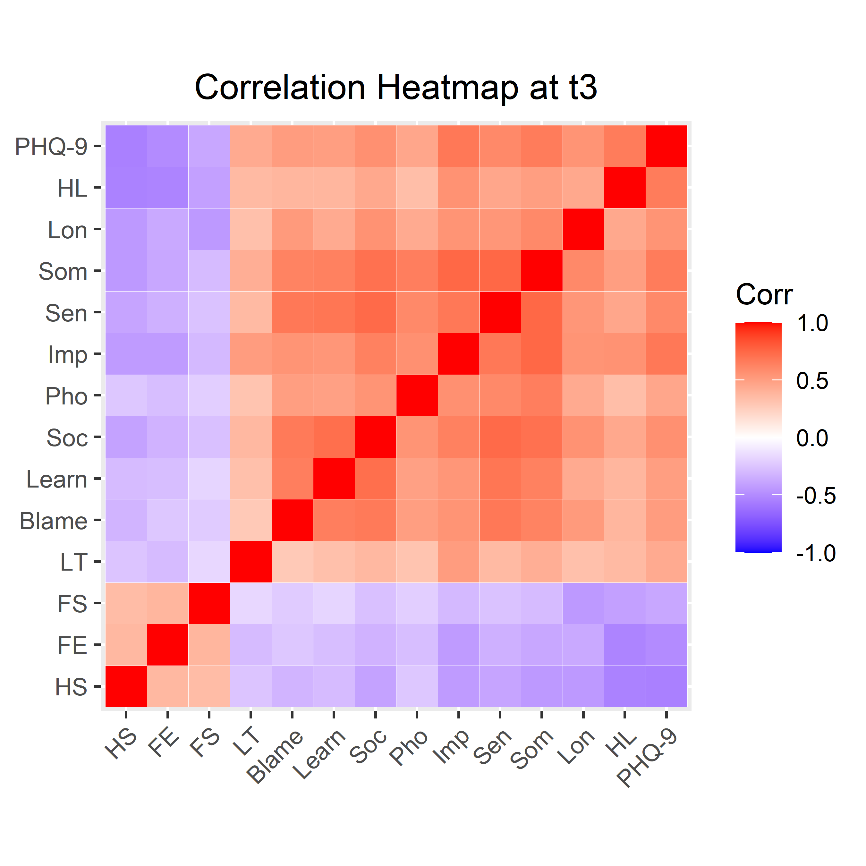

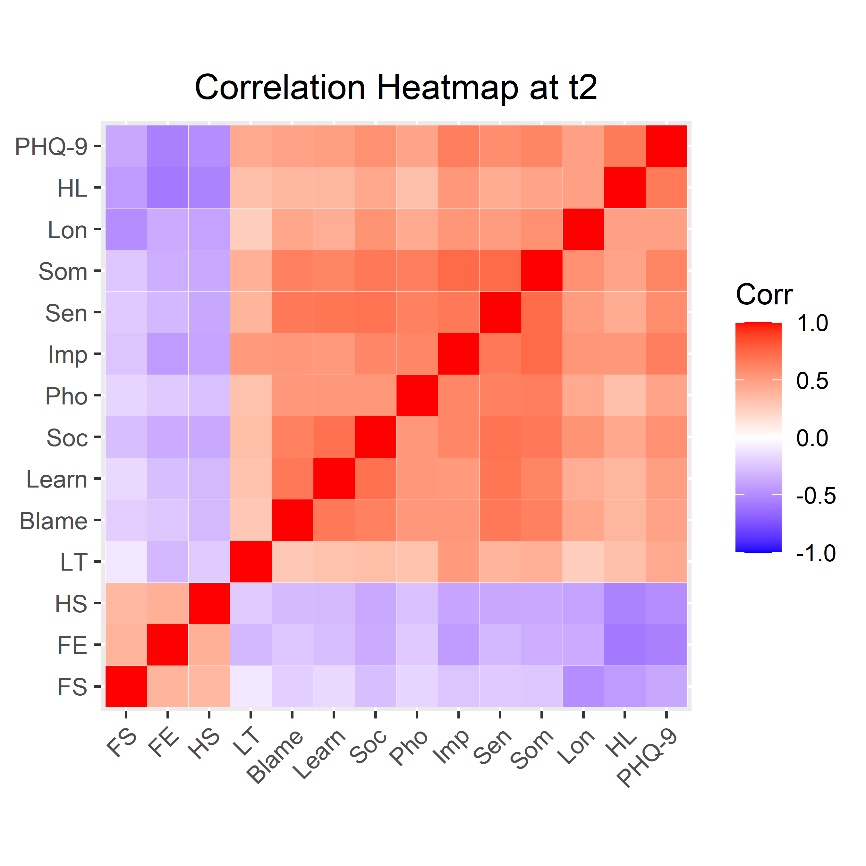

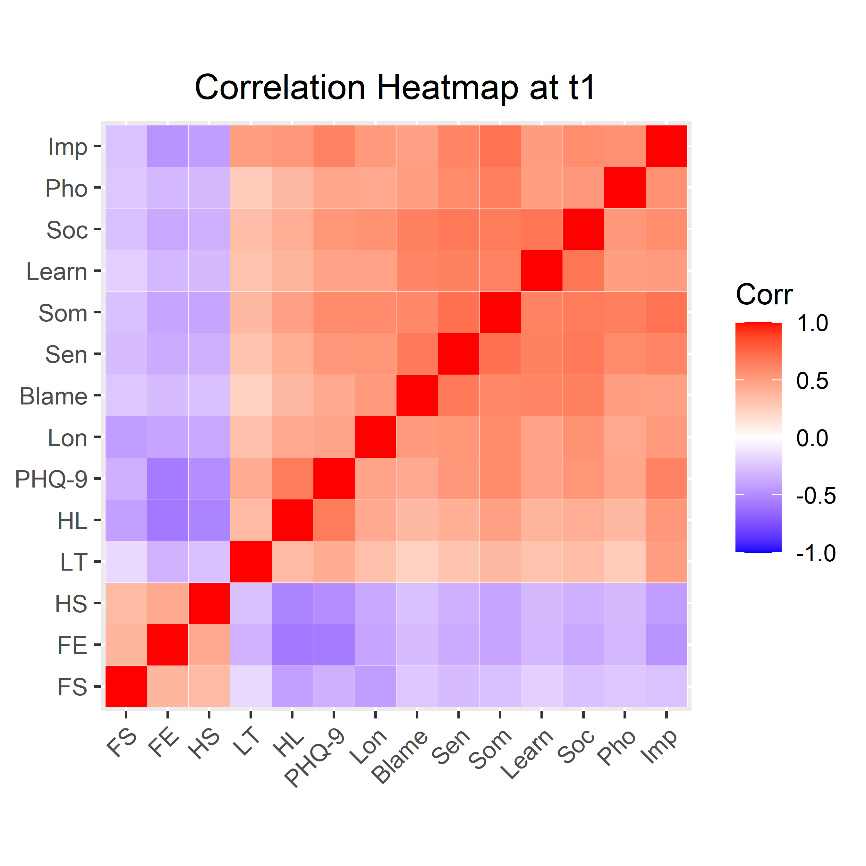

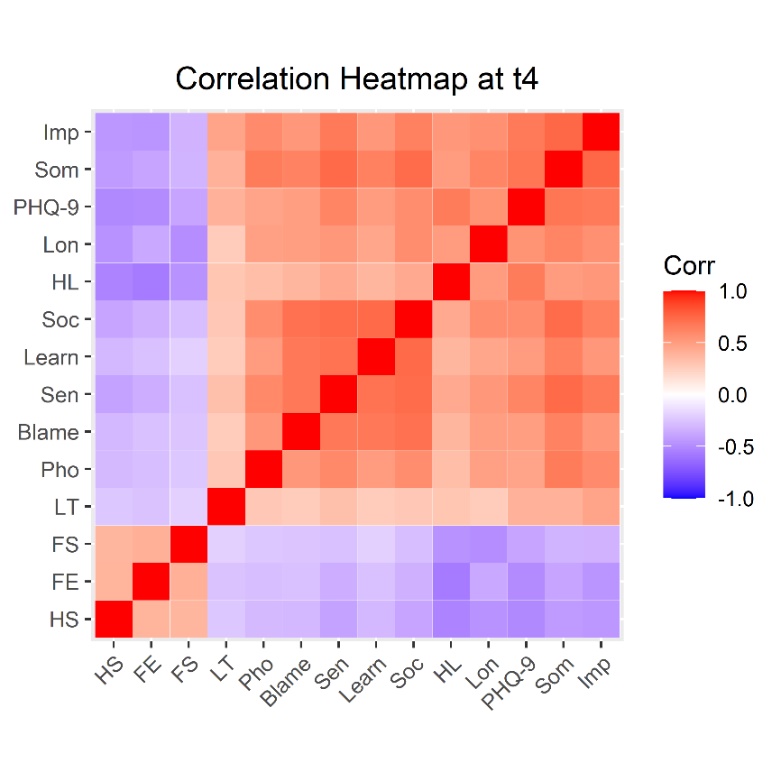


B

A

D

C

**Figure S2. Heatmap of correlation matrix across the entire sample at four time points**

The Pearson correlation coefficient matrix is expressed as a heatmap. 'Blame', 'FE', 'FS', 'HL', 'HS', 'Imp', 'Learn', 'Lon', 'LT', 'Pho', 'PHQ-9', 'Sen', 'Soc', 'Som' were 'Self-blaming', 'Family environment', 'Friendship', 'Hopelessness', 'Help-seeking', 'Impulsivity', 'Learning anxiety', 'Loneliness', 'Loss of temper', 'Phobia', 'PHQ-9 score', 'Oversensitivity', 'Social anxiety' and 'Somatic anxiety'. PHQ-9, the 9-item Patient Health Questionnaire.


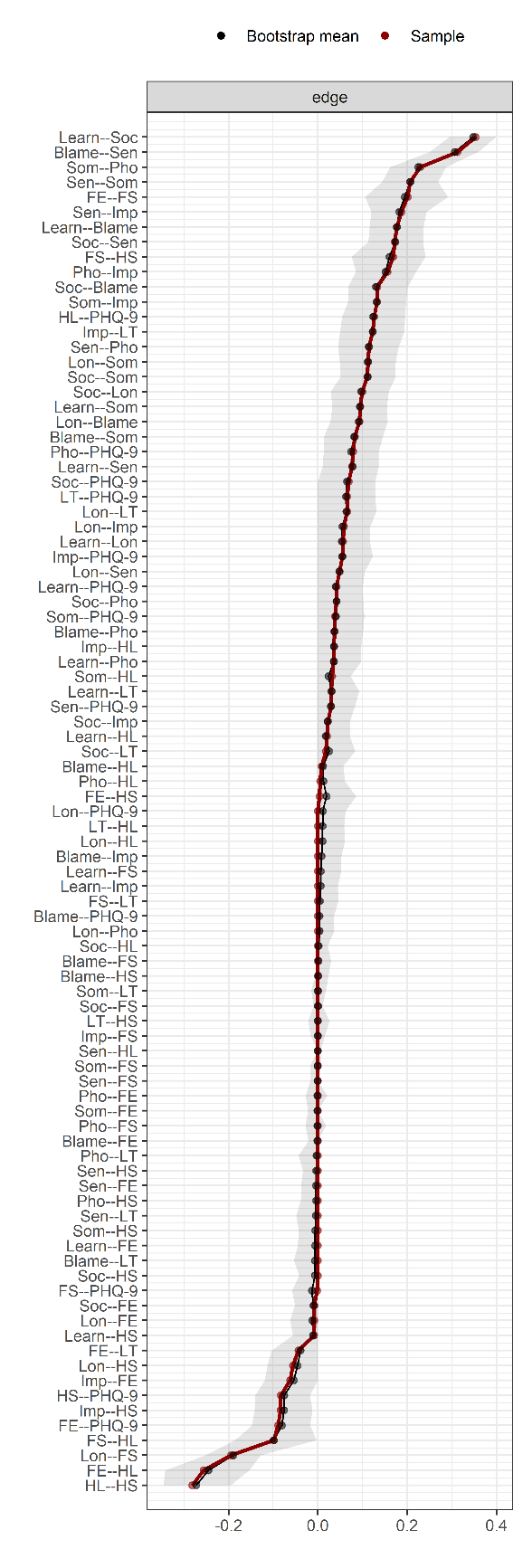

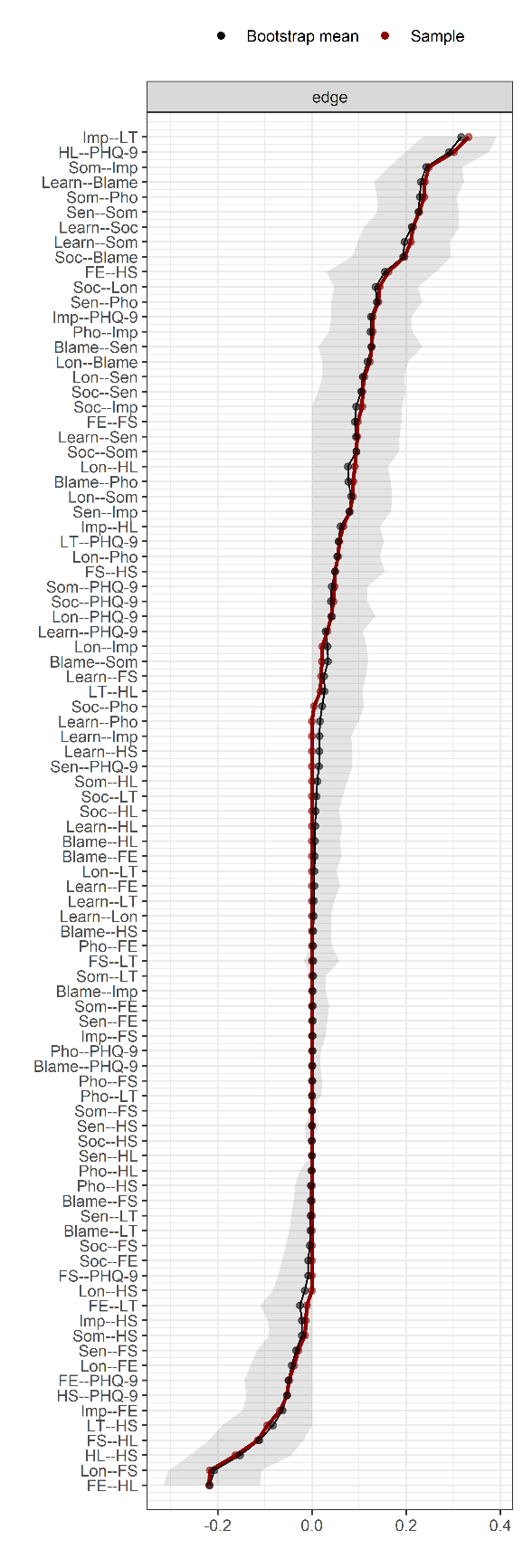


B

A

(A) The nondepressive symptom group at t1 (baseline). (B) The depressive symptom group at t1 (baseline).


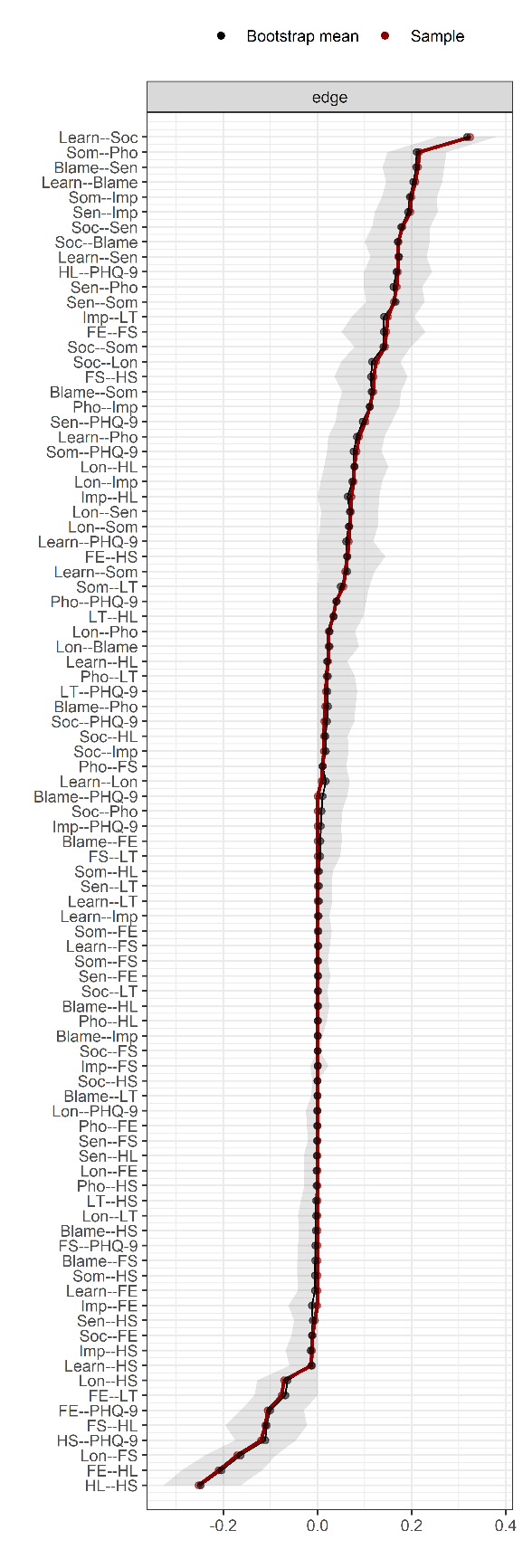

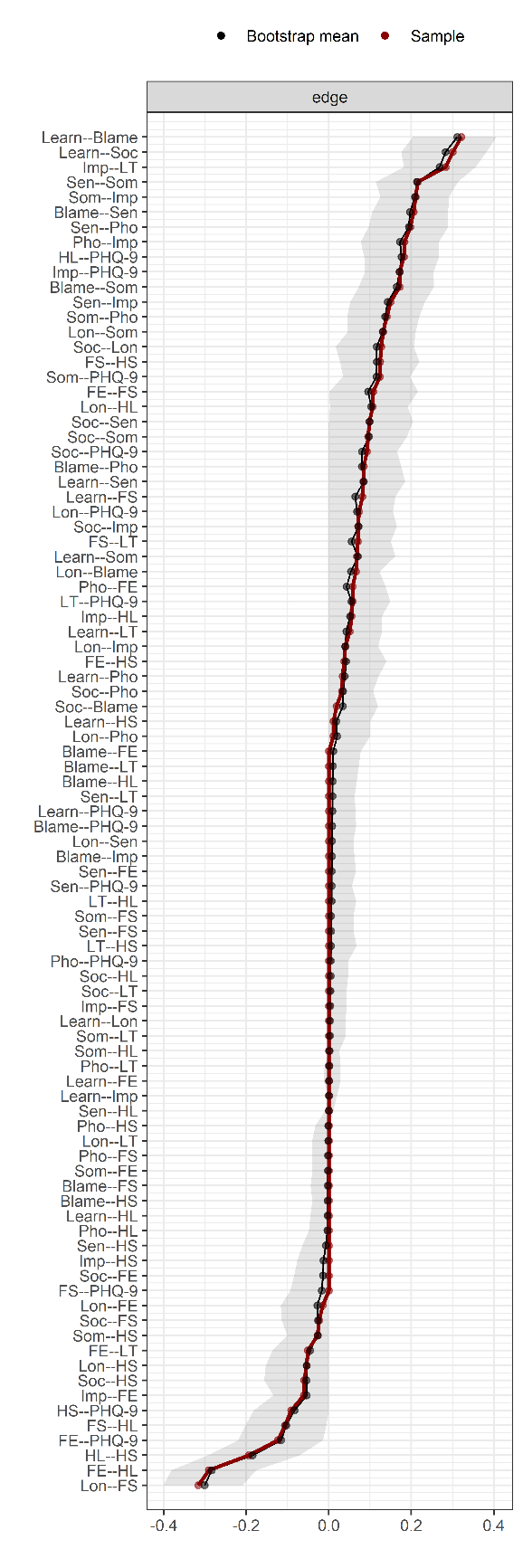


C

D

(C) The nondepressive symptom group at t2. (D) The depressive symptom group at t2.


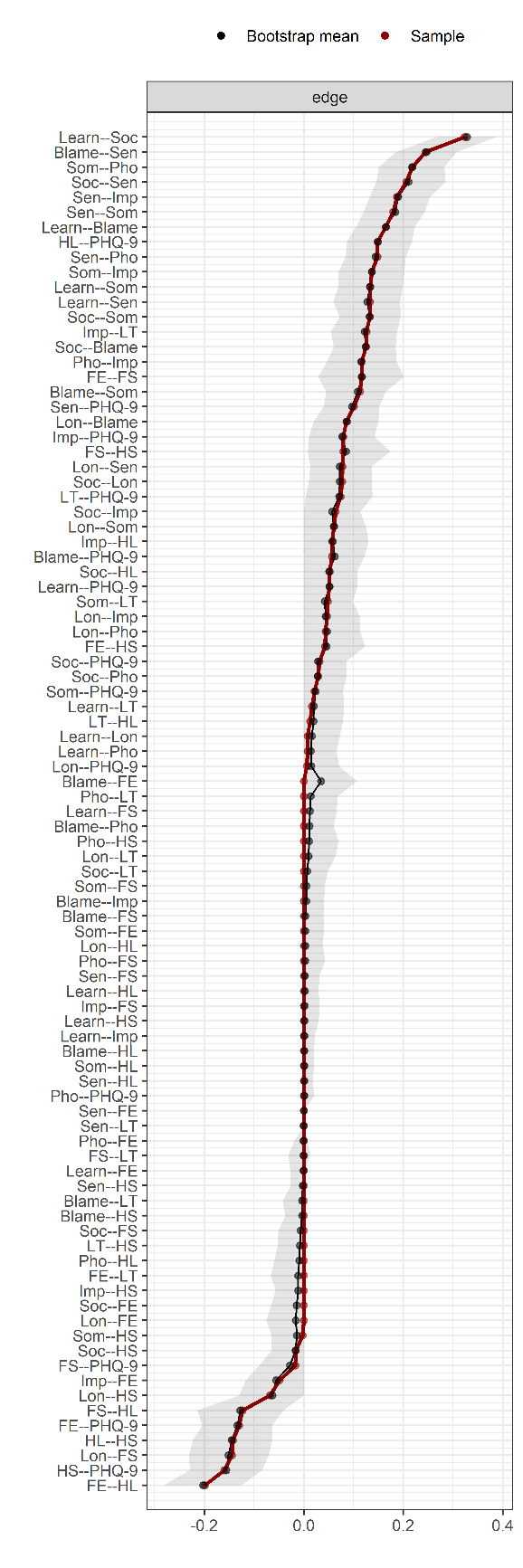

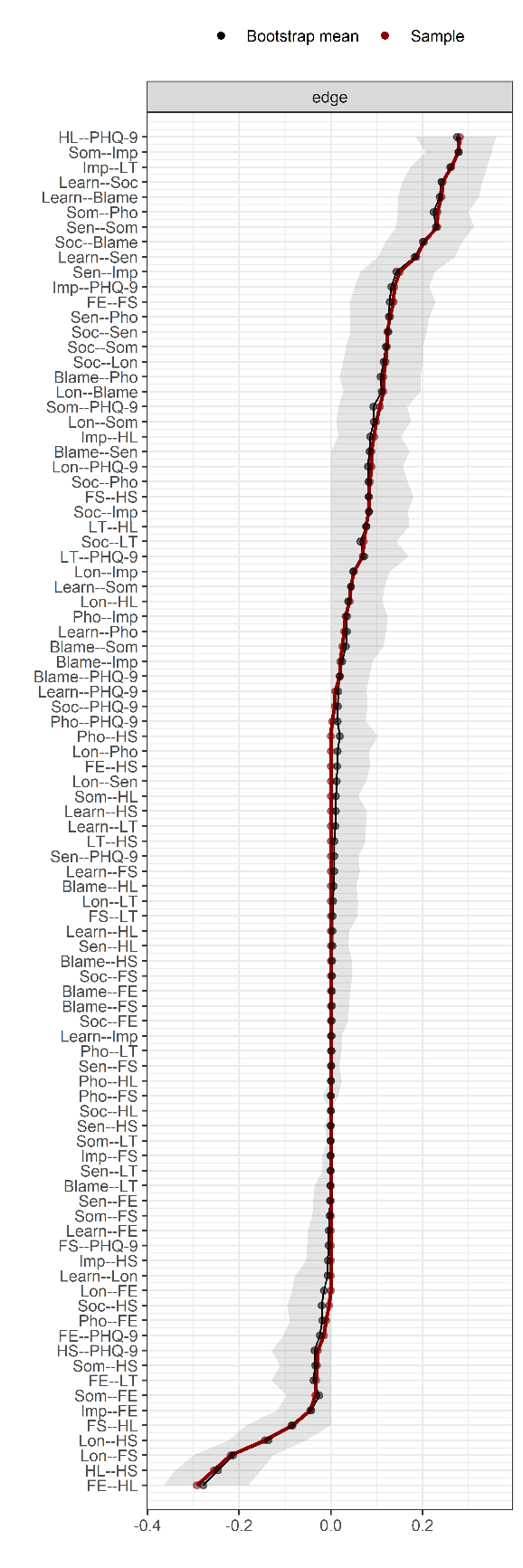


E

F

(E) The nondepressive symptom group at t3. (F) The depressive symptom group at t3.


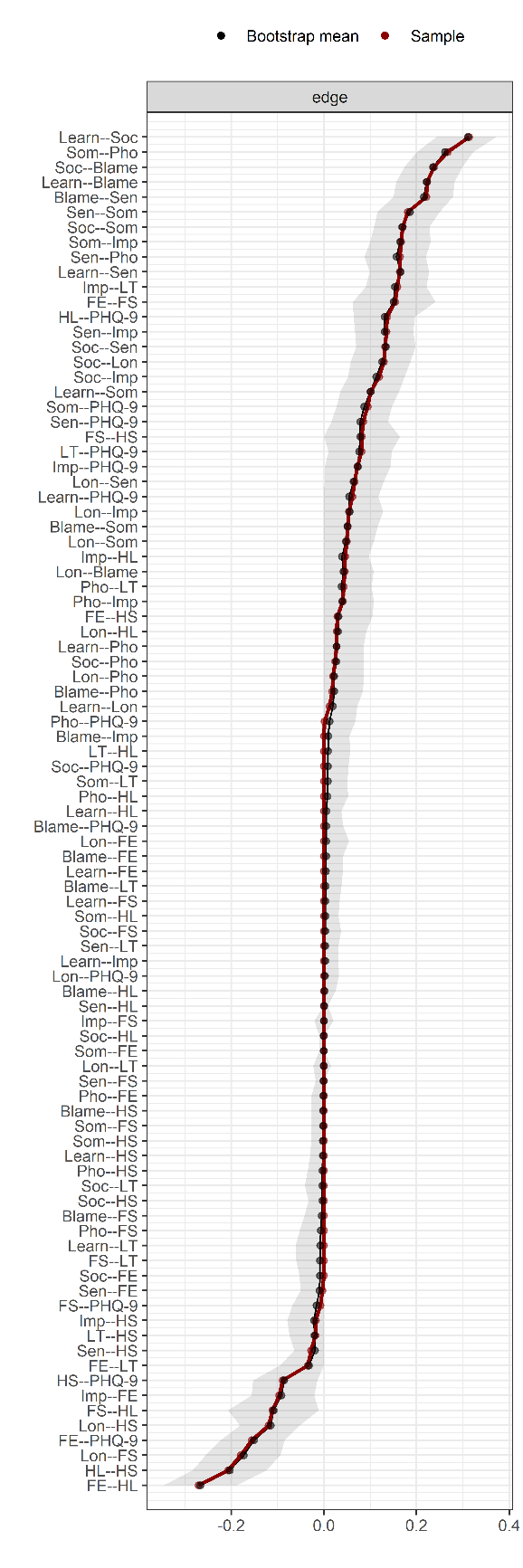

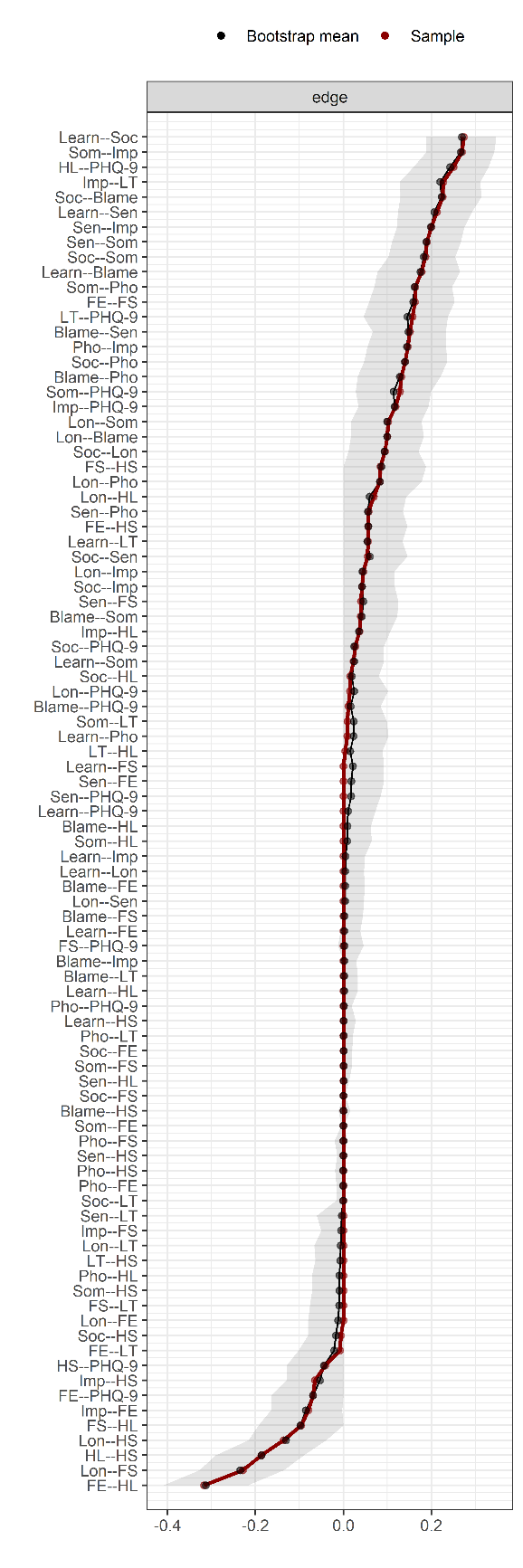


G

H

**Figure S3. Bootstrapped 95% confidence intervals (CI) for the estimated edge weights in the networks**

(G) The nondepressive symptom group at t4. (H) The depressive symptom group at t4. The red line indicates the sample values and the gray area the bootstrapped CIs. Each horizontal line represents one edge of the network, ordered from the edge with the highest edge-weight to the edge with the lowest edge-weight.


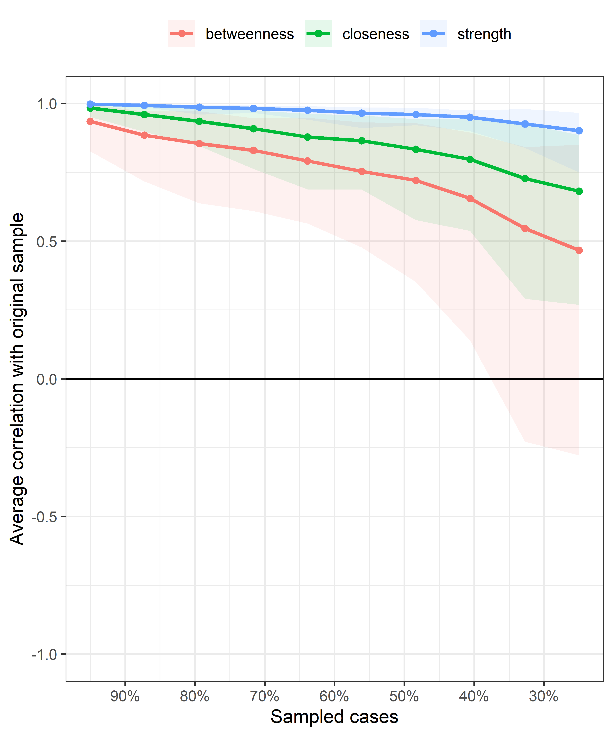

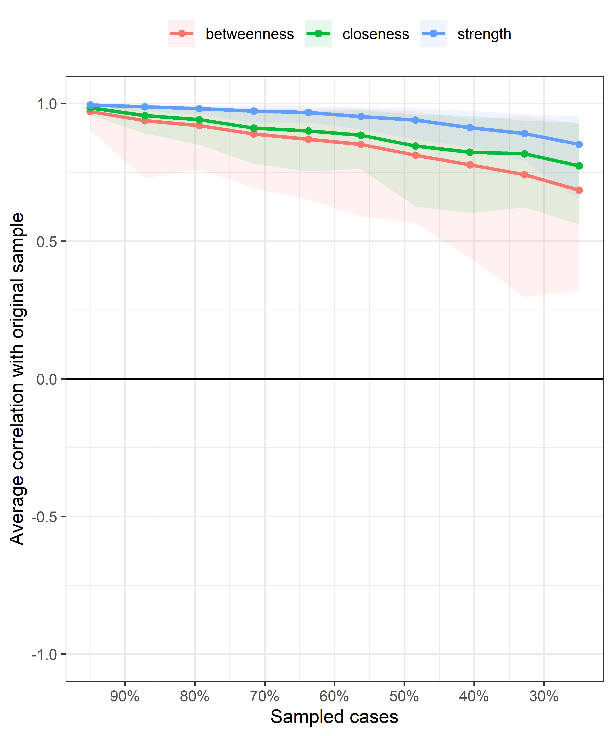


A

B


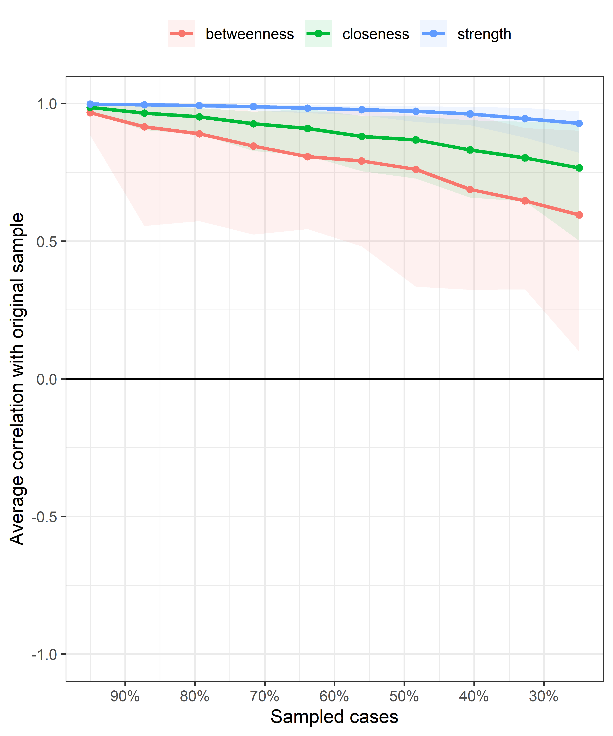

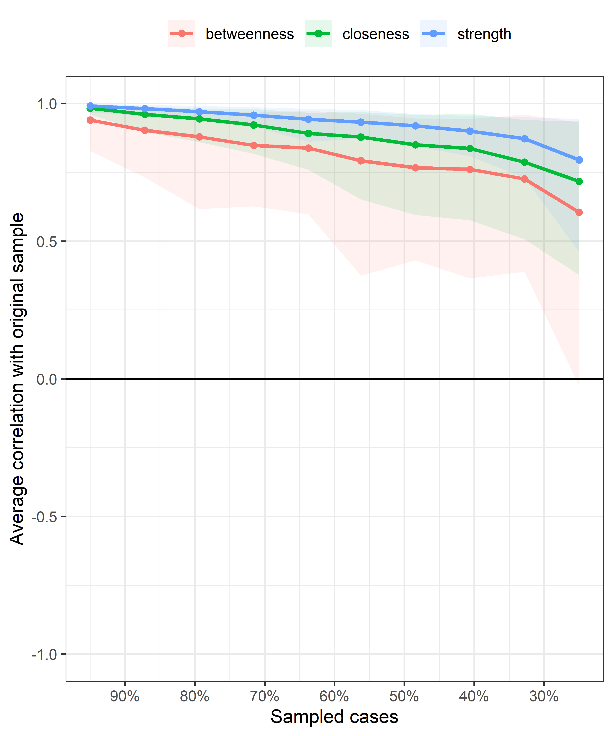


C

D

(A) The nondepressive symptom group at t1 (baseline). (B) The depressive symptom group at t1 (baseline). (C) The nondepressive symptom group at t2. (D) The depressive symptom group at t2. The x-axis represents the percentage of cases of original sample used at each step. The y-axis represents the average of correlations between the centrality indices from the original network and the centrality indices from the networks that were re-estimated after dropping increasing percentages of cases.


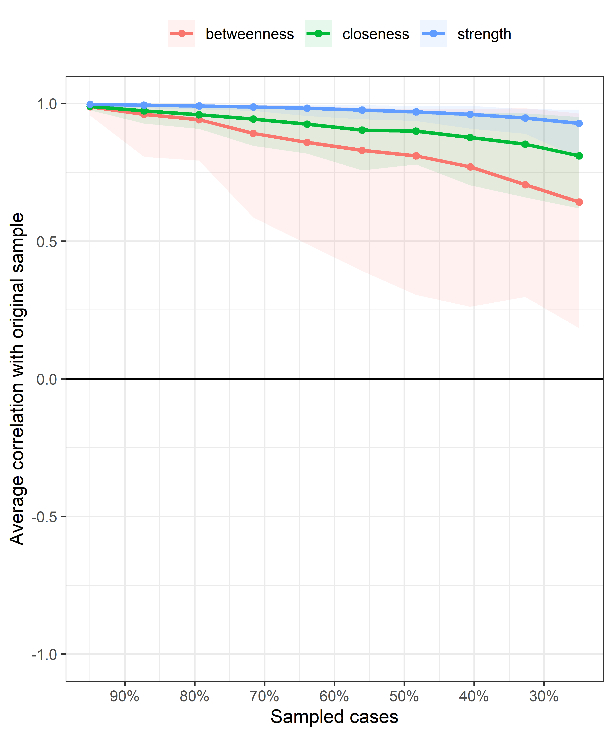

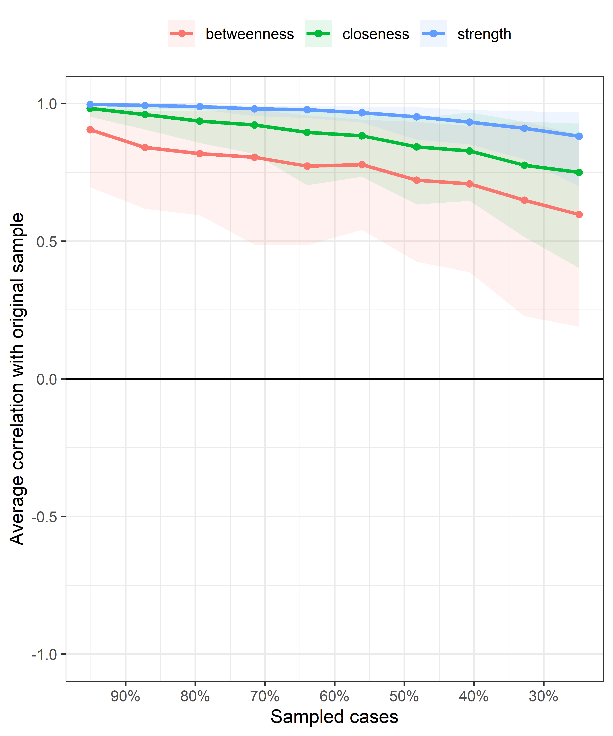


E

F


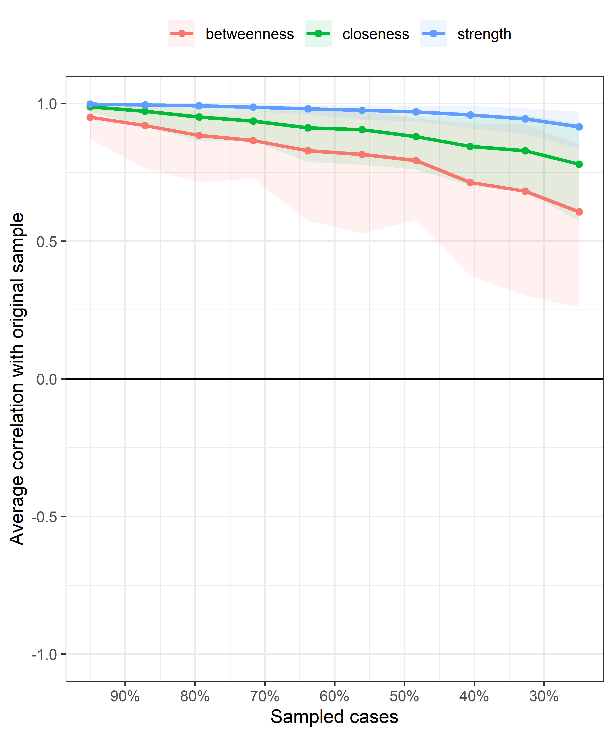

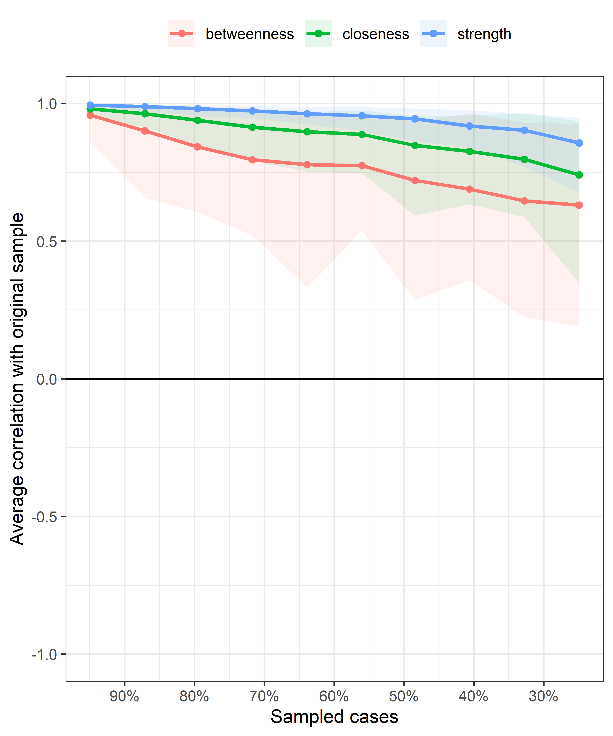


G

H

**Figure S4. Case-dropping bootstrap for the networks**

(E) The nondepressive symptom group at t3. (F) The depressive symptom group at t3. (G) The nondepressive symptom group at t4. (H) The depressive symptom group at t4. Average correlations between the centrality measures estimated with the full sample and the centrality measures estimated with smaller samples. Full line indicates the mean correlation while the colored area indicates the 2.5th quartile to the 97.5th quartile range.


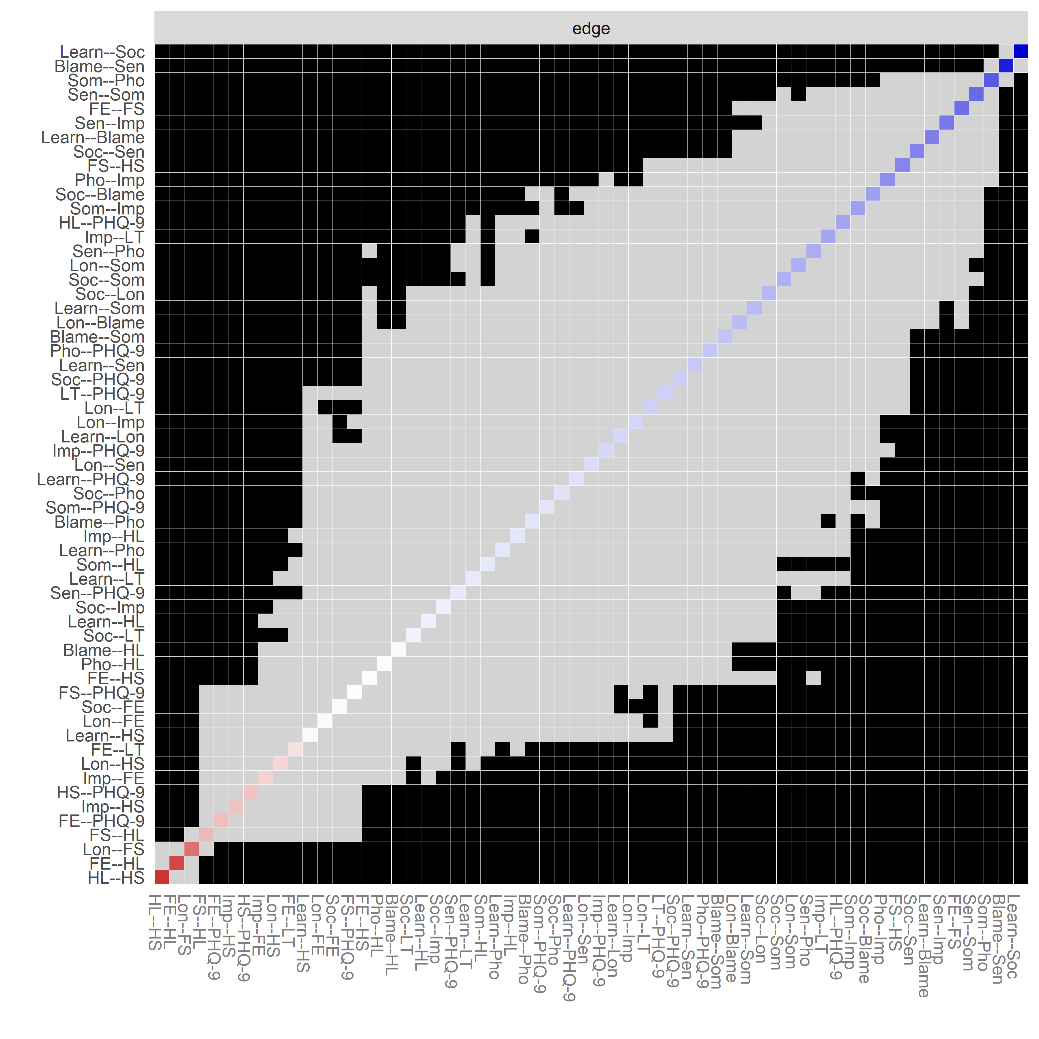


A


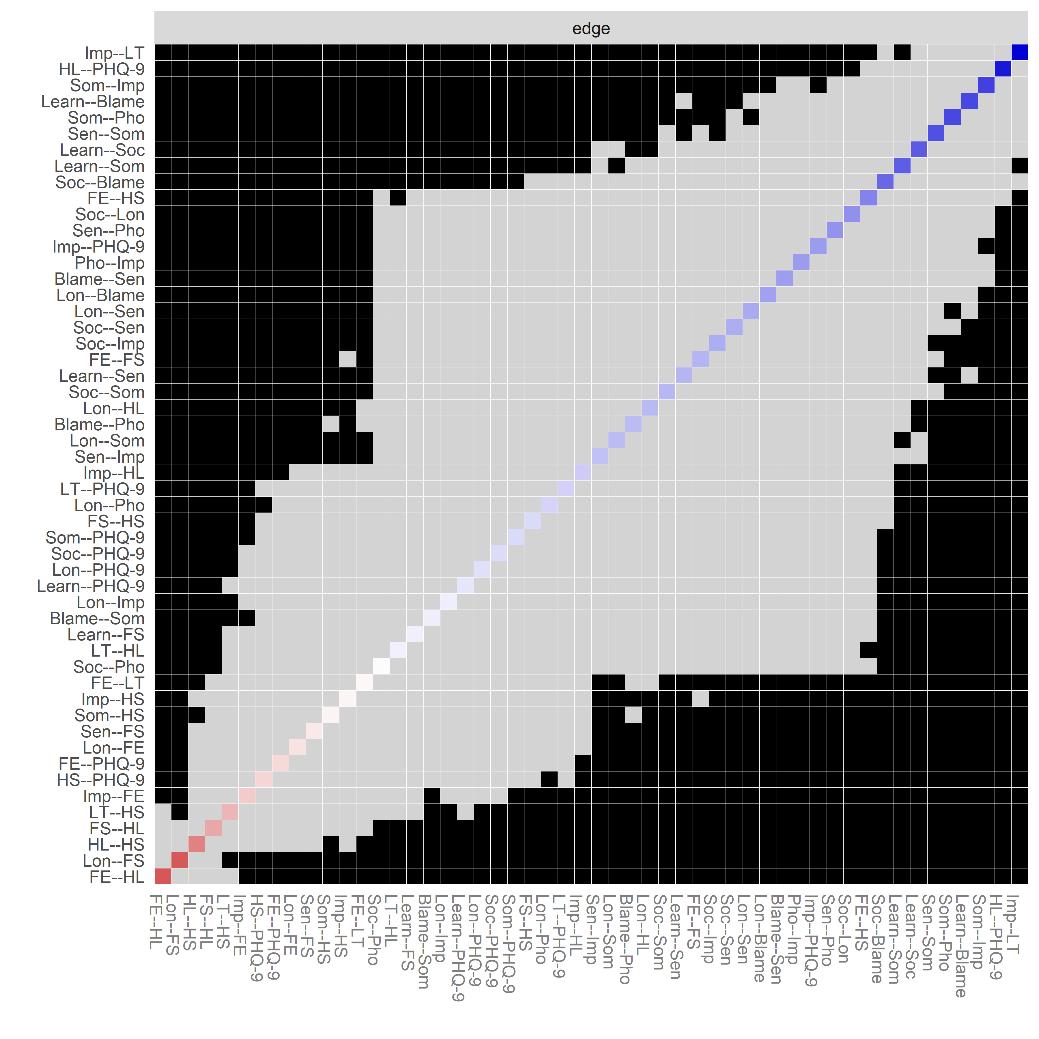


B


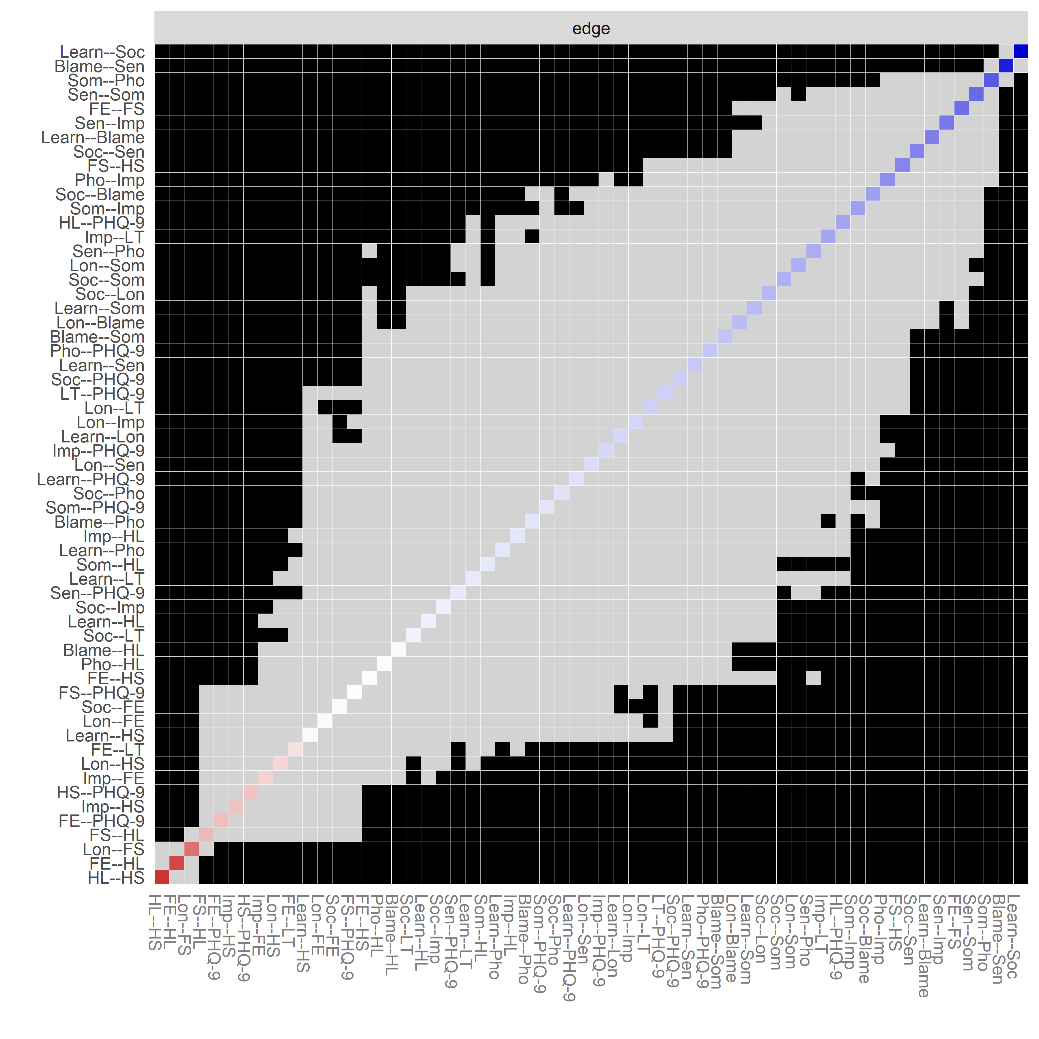


C

D


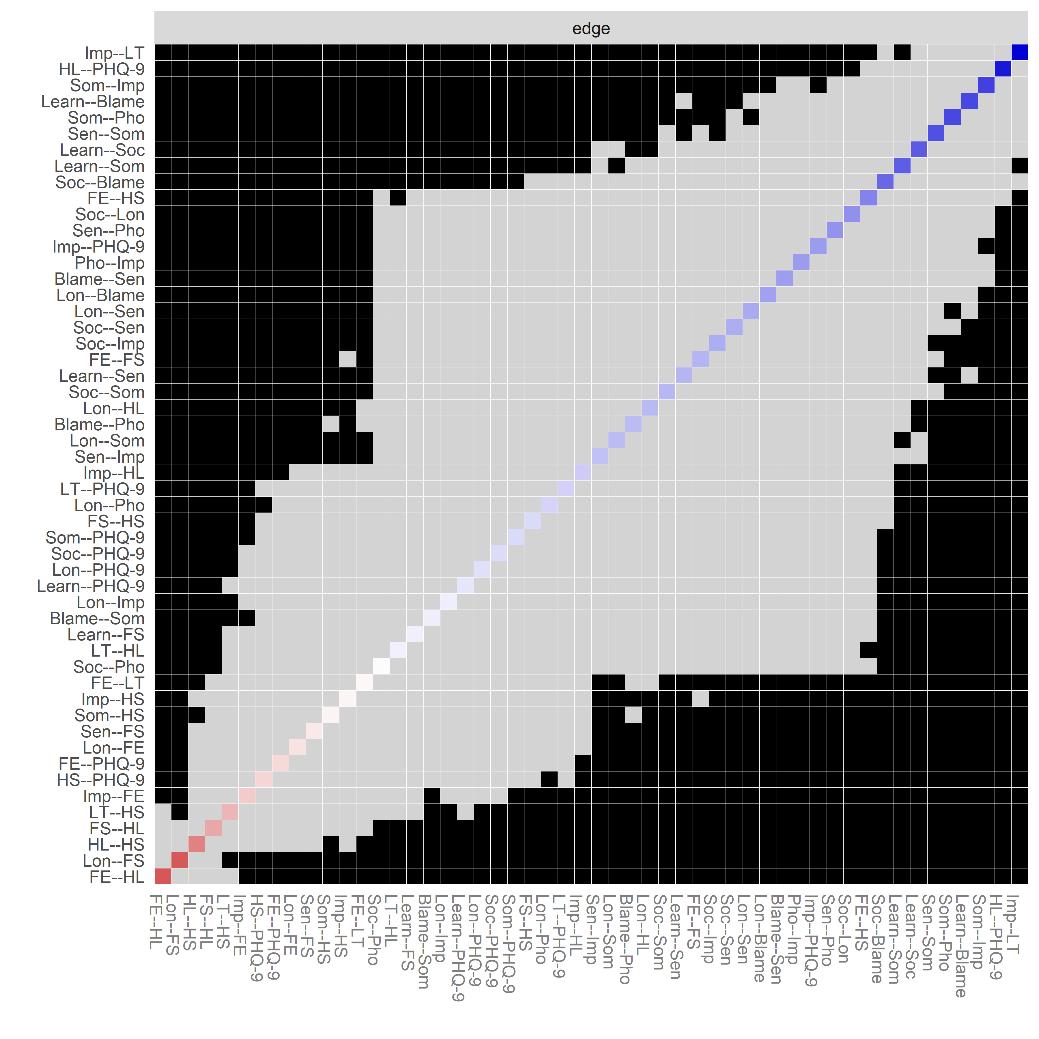


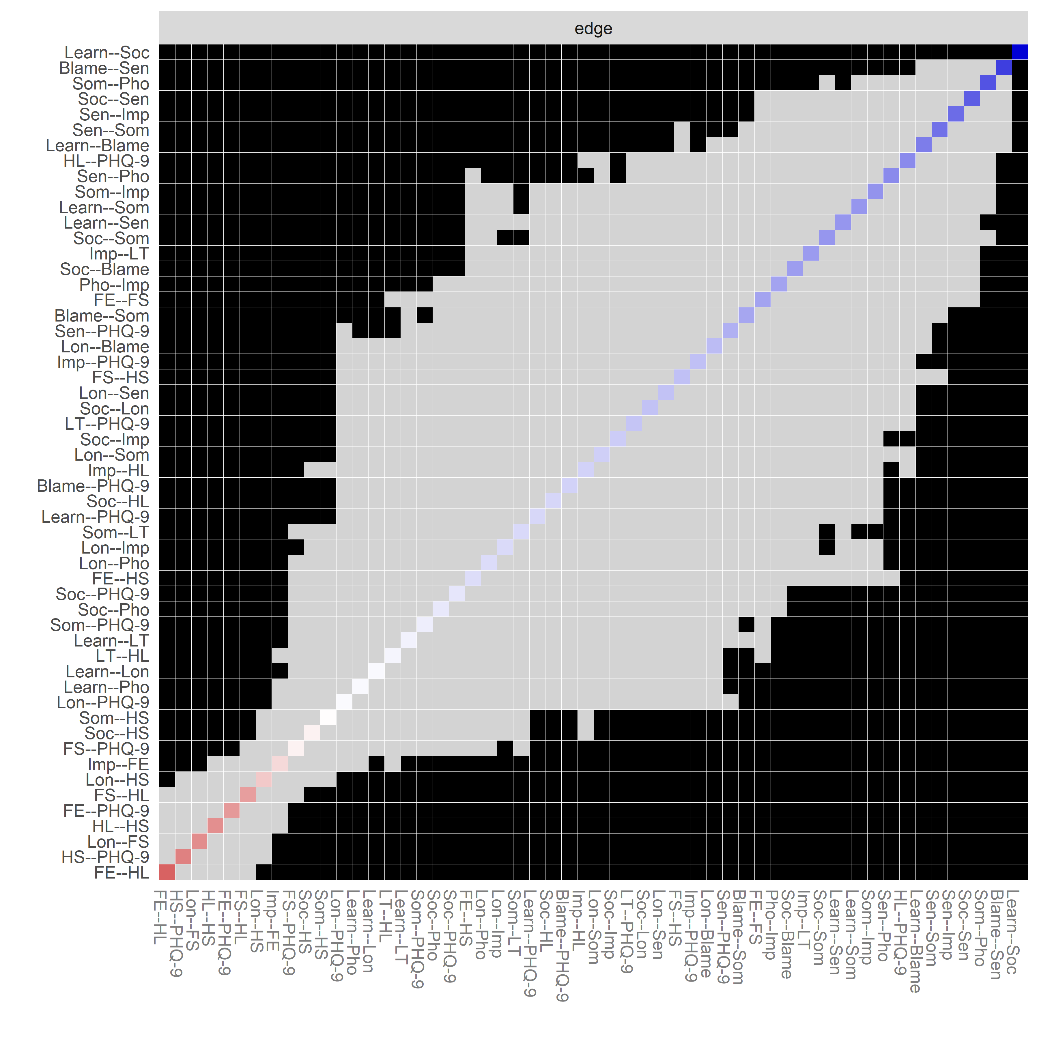


E

F


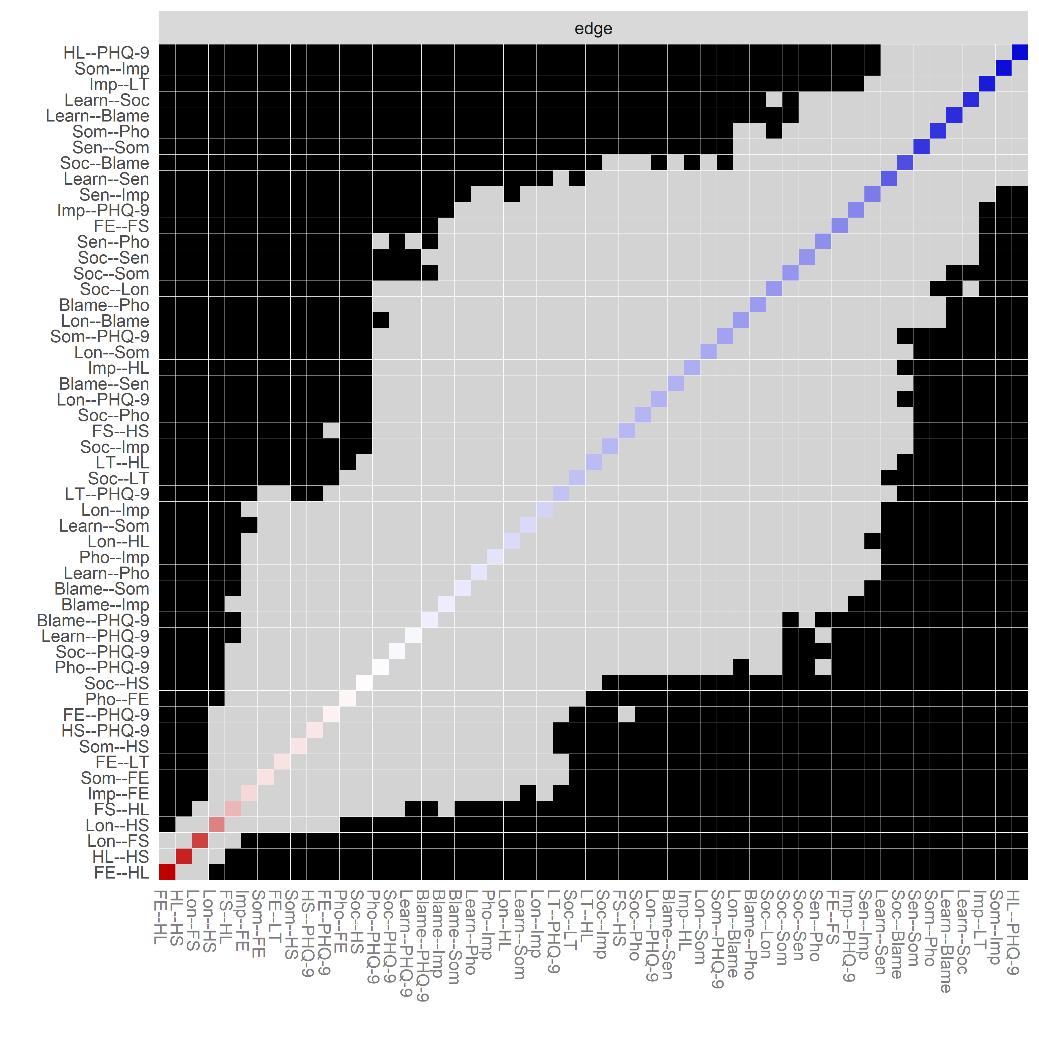


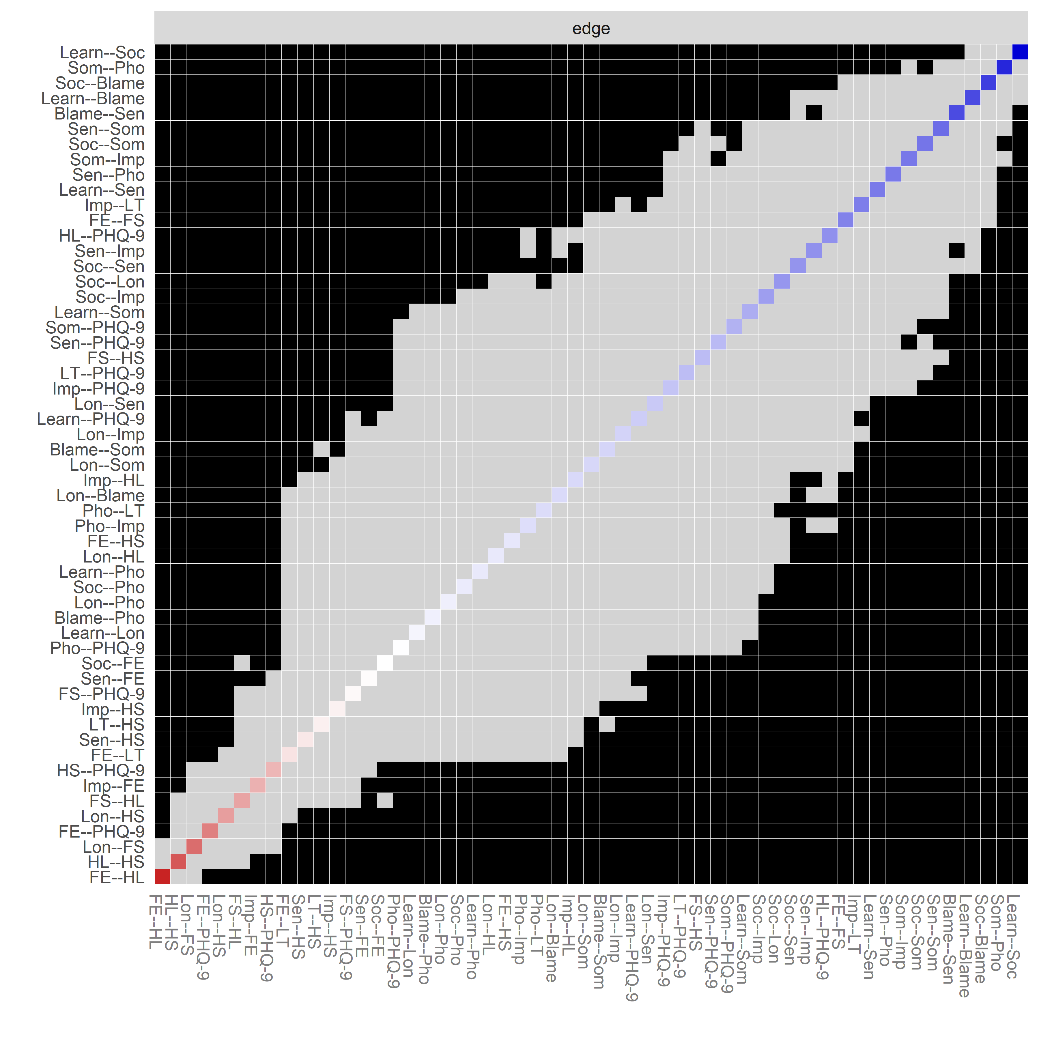


G

H


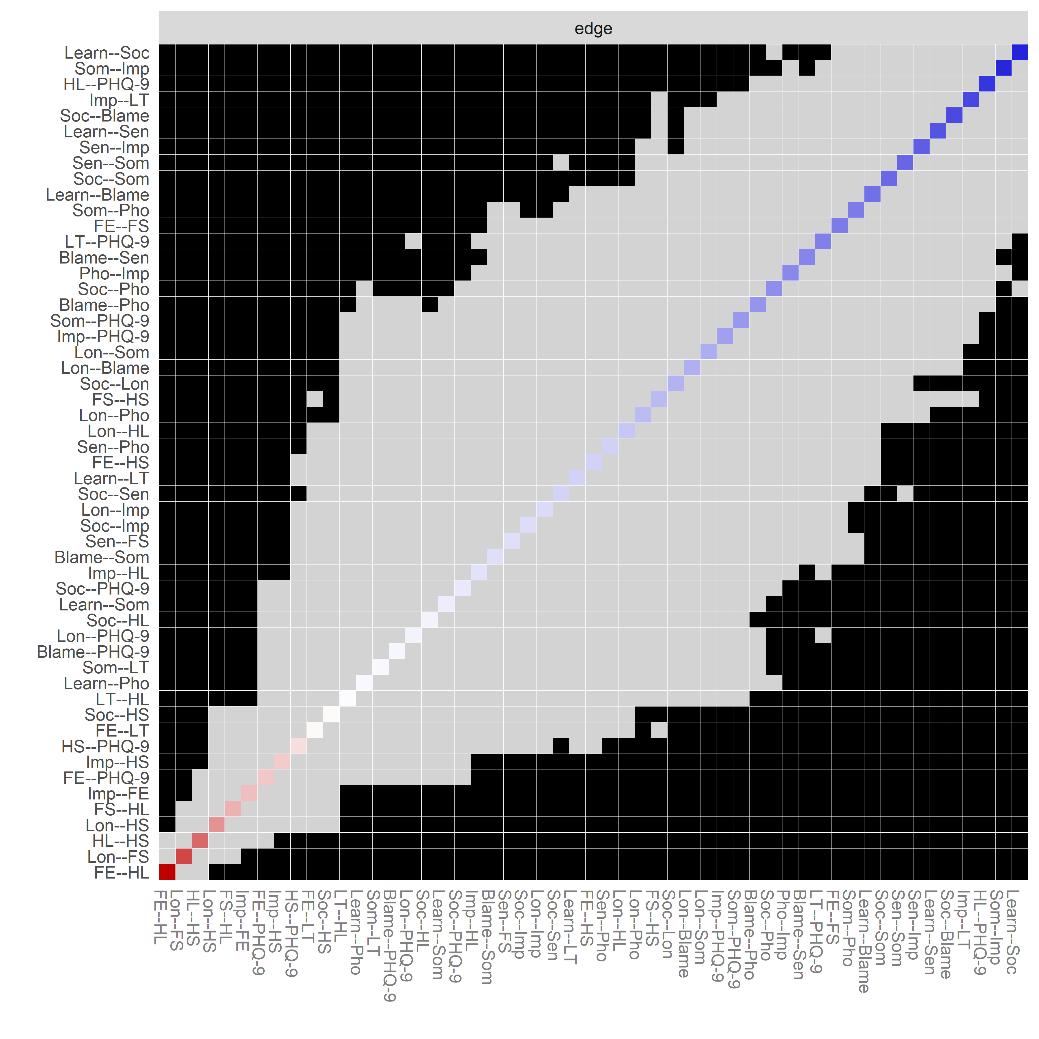


**Figure S5. Bootstrapped difference tests of edge weights in the networks**

(A) The nondepressive symptom group at t1 (baseline). (B) The depressive symptom group at t1 (baseline). (C) The nondepressive symptom group at t2. (D) The depressive symptom group at t2. (E) The nondepressive symptom group at t3. (F) The depressive symptom group at t3. (G) The nondepressive symptom group at t4. (H) The depressive symptom group at t4. Black boxes indicate a significant difference between two edges (alpha = .05). Grey boxes indicate no significant difference.


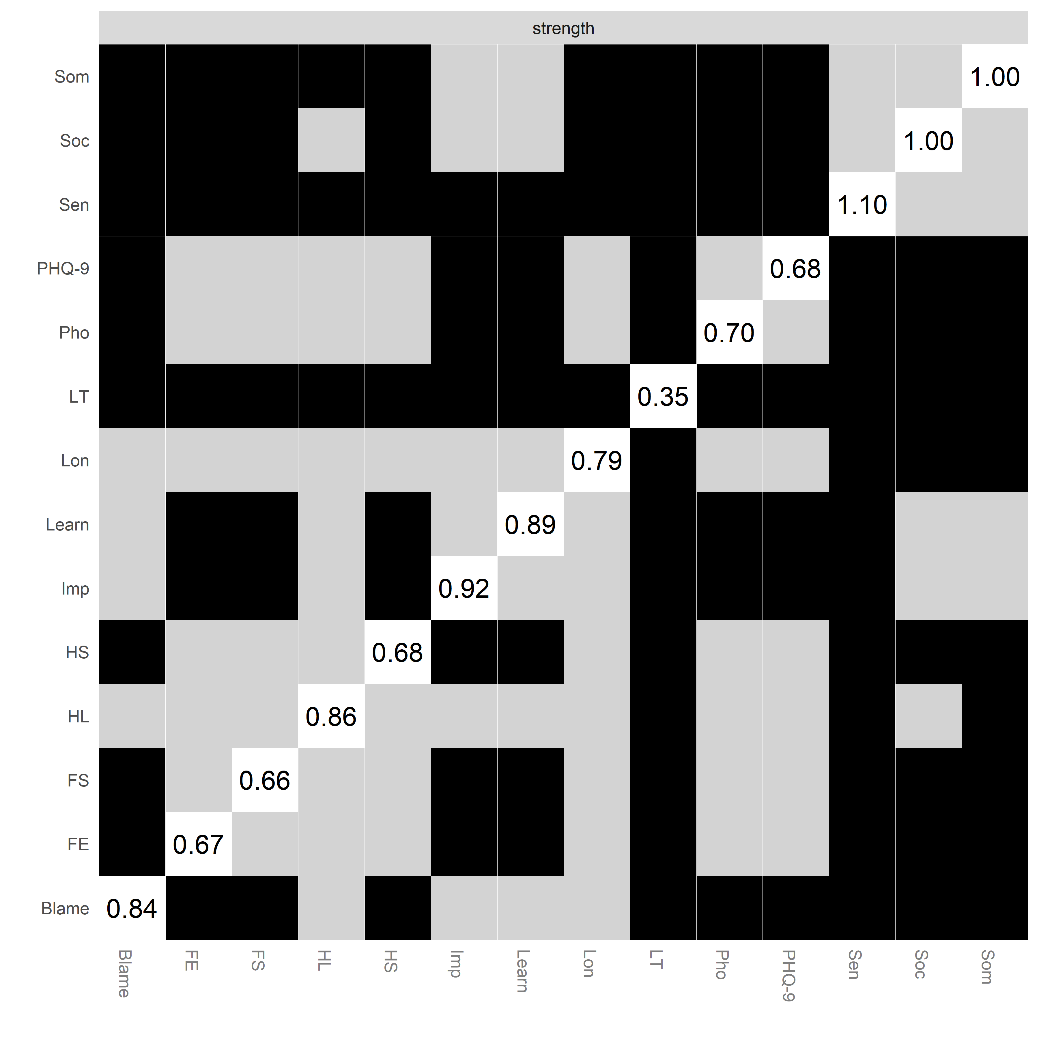


A

B


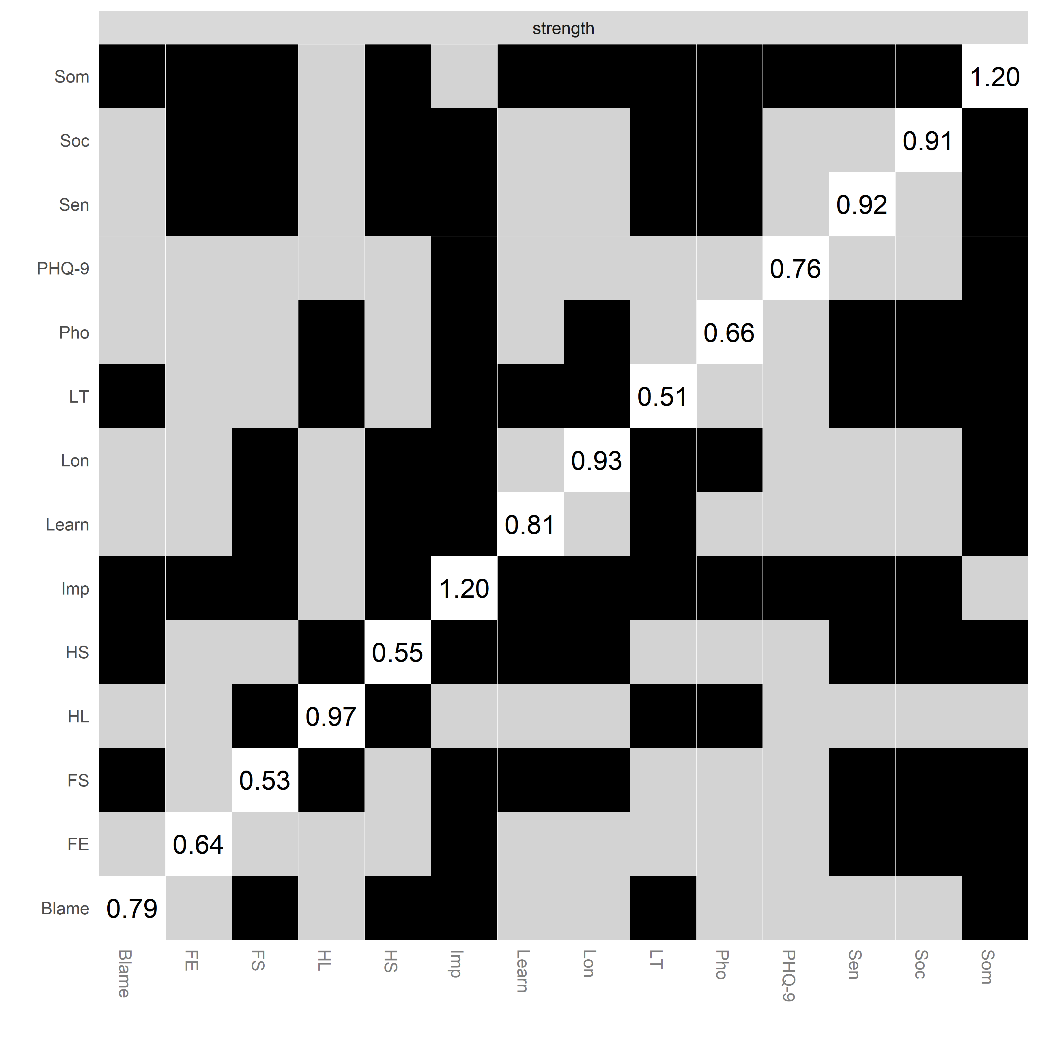


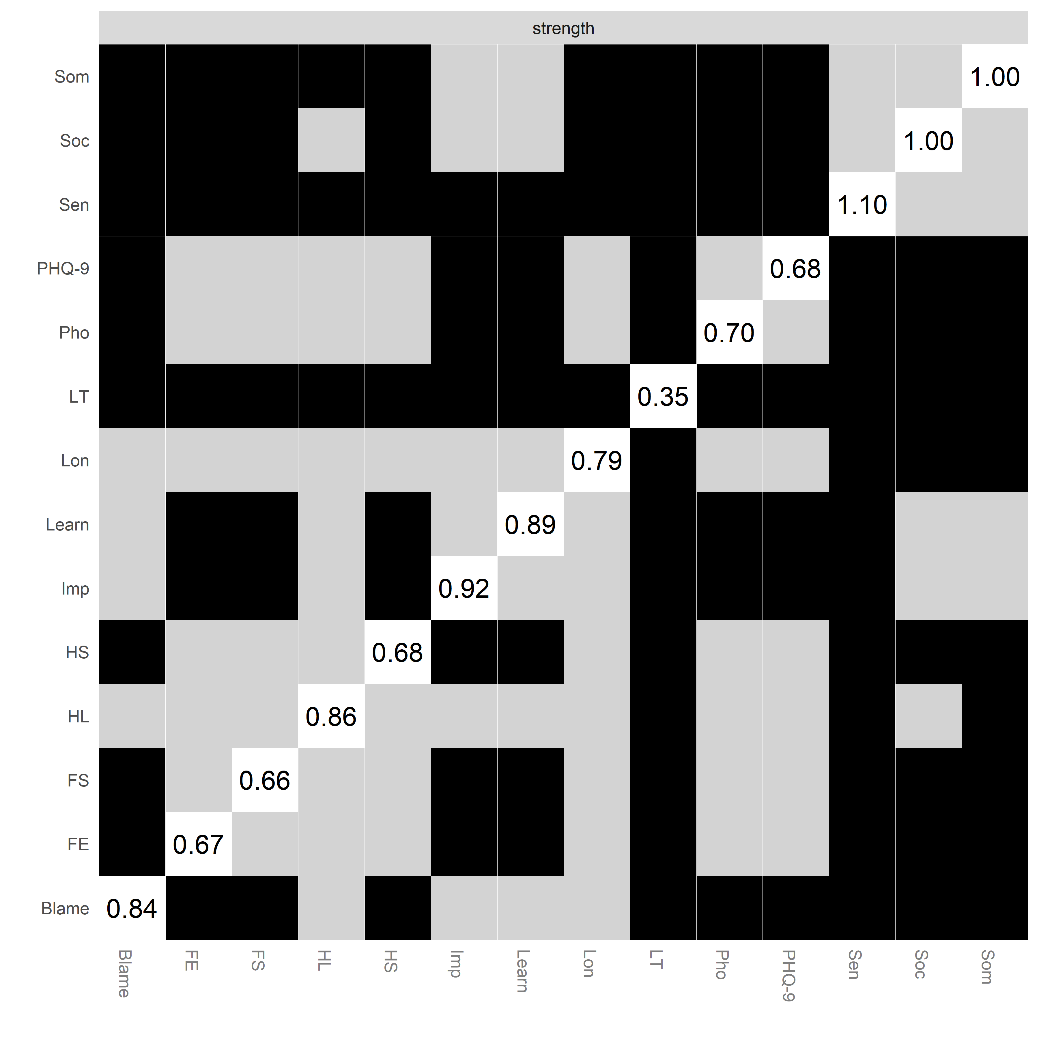


C

D


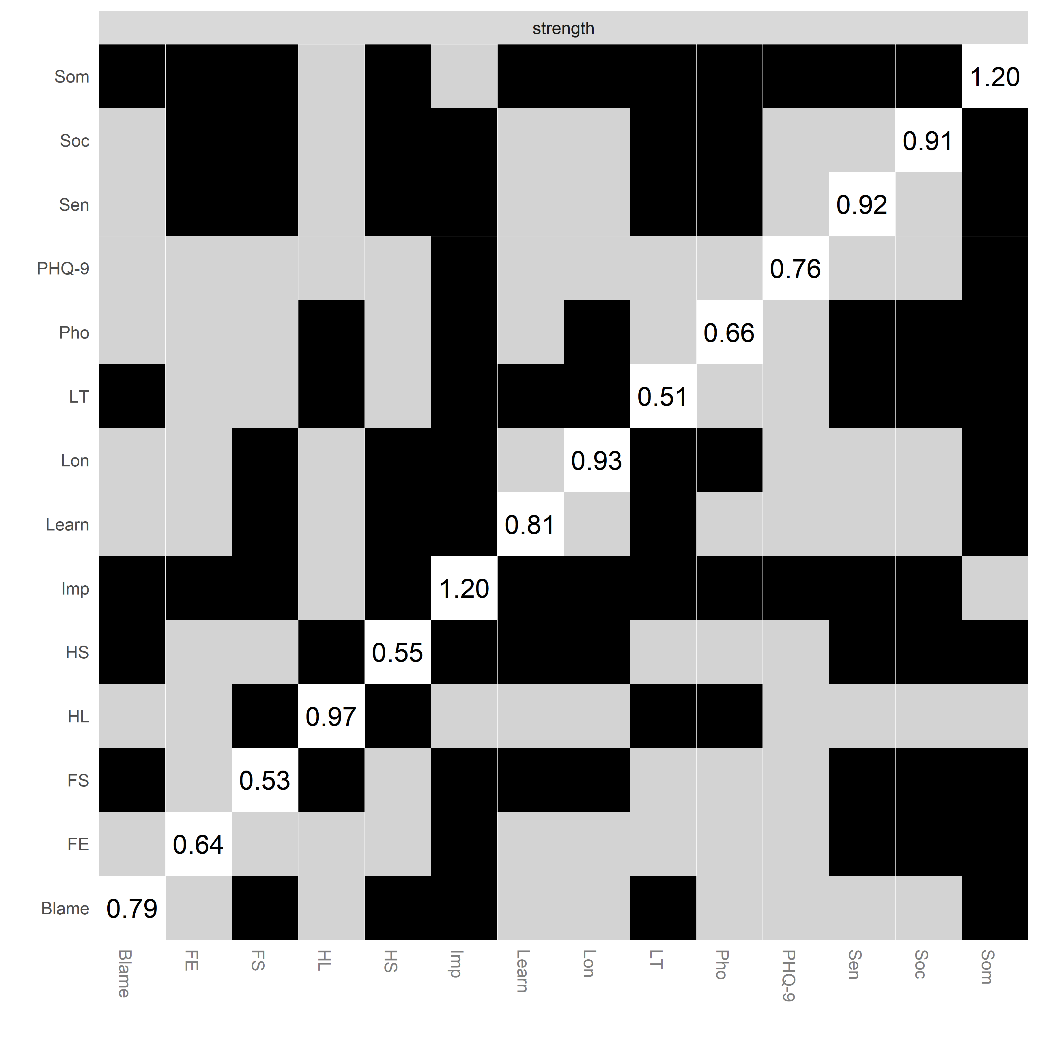


F

E


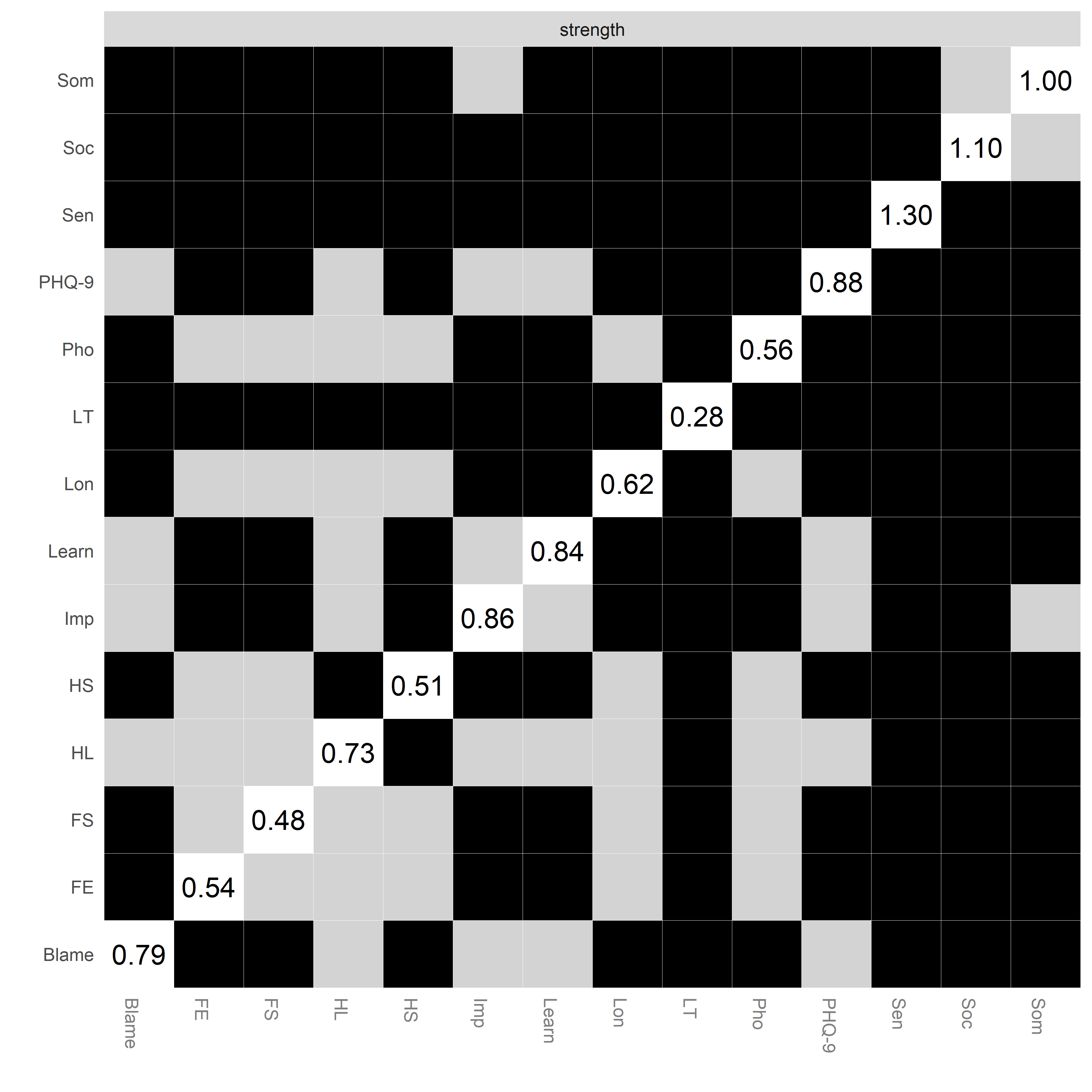


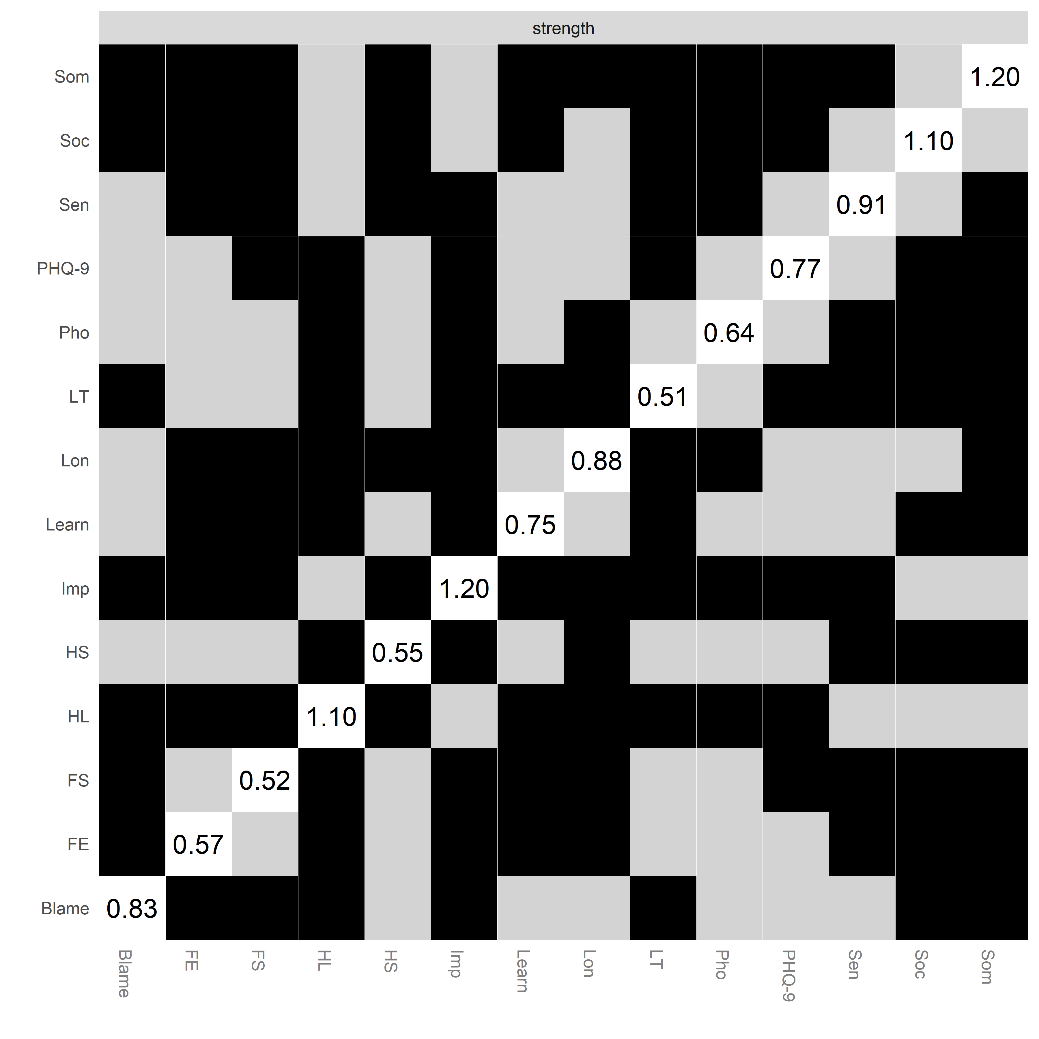


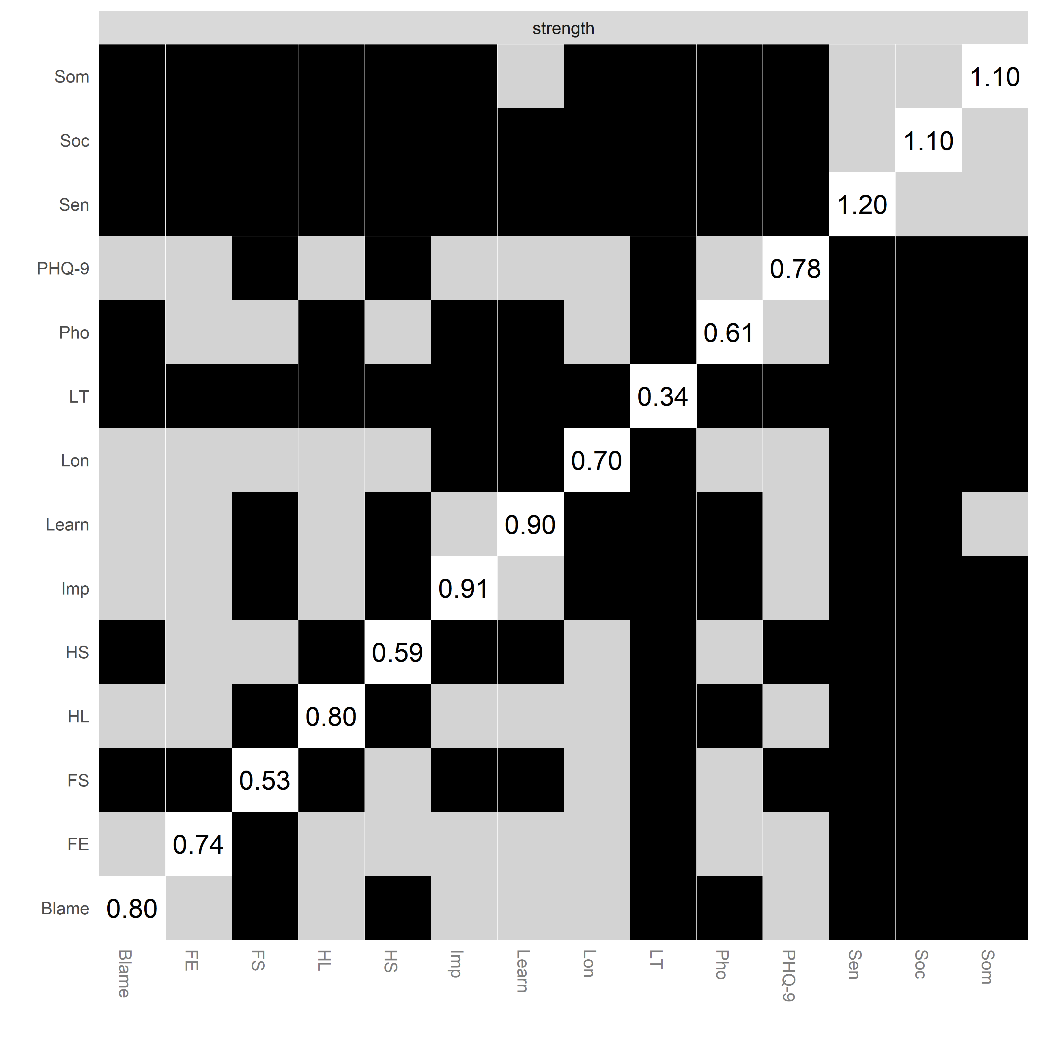


G

H


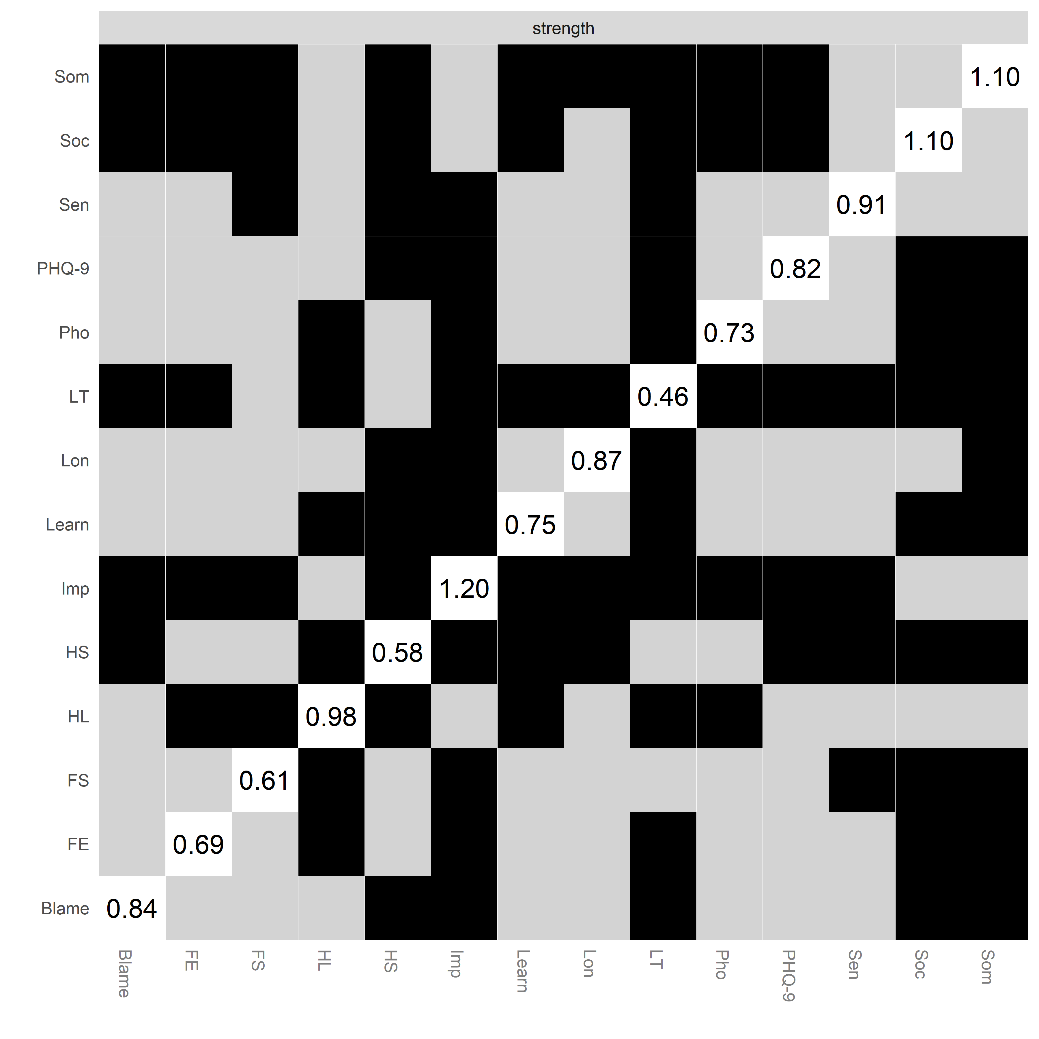


**Figure S6. Bootstrapped difference tests of strength centrality in the networks**

(A) The nondepressive symptom group at t1 (baseline). (B) The depressive symptom group at t1 (baseline). (C) The nondepressive symptom group at t2. (D) The depressive symptom group at t2. (E) The nondepressive symptom group at t3. (F) The depressive symptom group at t3. (G) The nondepressive symptom group at t4. (H) The depressive symptom group at t4. Black boxes indicate a significant difference between the strength centrality of two nodes (alpha = 0.05). Grey boxes indicate no significant difference.
